# Single‐Cell Profiling Across Immune Tissues and Organs Reveals Immunosenescence Signatures in Male Rhesus Monkeys

**DOI:** 10.1002/advs.202514353

**Published:** 2026-01-20

**Authors:** Shengnan Wang, Zhengna Zhu, Hongju Yang, Yaping Yan, Luyao Ran, Naixue Yang, Yichao Wang, Li Wu, Bing Bai, Weizhi Ji, Wei Si

**Affiliations:** ^1^ State Key Laboratory of Primate Biomedical Research Institute of Primate Translational Medicine Kunming University of Science and Technology Kunming Yunnan China; ^2^ Yunnan Key Laboratory of Primate Biomedical Research Kunming Yunnan China; ^3^ Geriatric Medical Center Division of geriatric Gastroenterology The First Afliated Hospital of Kunming Medical University Kunming Yunnan China

**Keywords:** immunosenescence, rhesus monkeys, single‐cell

## Abstract

Aging gradually impairs immune system function, yet its systemic features across immune organs remain poorly characterized in primates. Here, we perform single‐cell transcriptomic profiling of bone marrow, spleen, mesenteric lymph nodes, and peripheral blood mononuclear cells from young and naturally aged male rhesus monkeys. Our study revealed extensive transcriptional remodeling across tissues, particularly the marked upregulation of GZMB expression across multiple cell types in aged monkeys, highlighting it as a candidate biomarker of immunosenescence. Gene regulatory network analysis identifies BHLHE40 as a key transcription factor enriched in multiple CD8^+^ T cell subtypes during aging, regulating pro‐inflammatory and exhaustion‐related genes. We also observe an age‐associated expansion of CD8^+^ central memory T cells with increased CCL5 and reduced IL7R expression, consistent with a shift toward a dysfunctional state. In the bone marrow, we discover a distinct naïve B cell population with low PDCD4 expression that declines with age, potentially compromising humoral immunity. These findings offer a comprehensive single‐cell atlas of immune aging in a non‐human primate model, providing novel insights into cell‐type‐specific and tissue‐dependent features of immunosenescence. Our work establishes a valuable resource for future translational studies and biomarker discovery in human aging.

## Graphical abstract

Single‐cell profiling across bone marrow, spleen, mesenteric lymph, and blood in rhesus monkeys reveals organ Immunosenescence. GZMB rises with age, particularly in cytotoxic and terminally exhausted CD8^+^ T cells, and BHLHE40 emerges as a key transcription factor enriched across multiple CD8^+^ subsets, regulating pro‐inflammatory and exhaustion‐related genes; these patterns replicate in human bone marrow and PBMCs. PDCD4^low^ naïve B cells decline in bone marrow, and Mesenteric lymph cDC2 cells show reduce antigen presentation. These findings highlight candidate biomarkers and regulatory nodes for intervention.

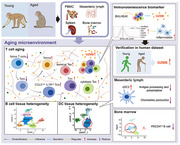

## 1 Introduction

Aging is characterized by the progressive loss of physiological integrity, which leads to impaired functions, and represents the greatest cause of disease and death globally [1, 2]. Aging implies a complex array of changes and remodeling in homeostatic mechanisms that regulate the immune system, encompassing both quantitative and functional changes in various cellular subtypes. Immune system deterioration has become one of the hallmarks of aging [3]. Understanding the underlying mechanisms driving age‐related immune dysfunction, exploring immunosenescence makers, and developing antiaging strategies have therefore become urgent global priorities.

A comprehensive single‐cell transcriptional atlas encompassing multiple immune organs and tissues in humans will greatly increase our understanding of the cellular characteristics and regulatory networks underlying immunosenescence. Recently, single‐cell transcriptome profiling of 15 major organs has been reported for a single adult human, but without the involvement of aging [4]. Currently, the majority of studies focusing on the progressive loss of healthy physiology during aging have focused mainly on rodent [5], and recent studies have reported that cell‐to‐cell transcriptional variability increases in CD4^+^ T cells during immunosenescence [6]. Moreover, aging promotes the accumulation of *PD1* ^+^ *GZMK* ^+^CD8^+^ T cells in mice [7, 8]. However, there are significant differences between the mouse and human immune systems. The production of human T cells depends on the peripheral proliferation of naïve T cells [9]. In contrast, mouse naïve T cells are completely dependent on thymus activity [10, 11, 12]. Therefore, it is notoriously difficult to translate rodent immunological principles to humans because of the fundamental immunological disparities between the two species [13, 14]. Furthermore, ethical challenges associated with the acquisition of human samples have predominantly directed investigations on human immunosenescence toward peripheral blood. Peripheral blood contains less than 3% of the total T cells in the body and therefore does not fully reflect the changes associated with the aging human immune system [15]. Consequently, a well‐defined human immunosenescence atlas comprising various cell types from multiple tissues and organs has not yet been established.

The rhesus monkey is one of the most widely used animal models in biomedical research and is phylogenetically closely related to humans [16, 17]. In addition, the immune‐associated gene expression patterns and cellular communications in rhesus monkeys are strikingly more similar to humans than those in mice [18]. Therefore, in this study, we utilized four immune tissues and organs, including hematopoietic tissue (bone marrow), immune lymph tissue (mesenteric lymph), peripheral blood mononuclear cells (PBMC), and immune organ (spleen) samples, from young and naturally aged monkeys to investigate the dynamic changes and regulatory networks associated with primate immunosenescence.

## 2 Experimental Section

### 2.1 Animals and Ethics

Two male rhesus monkeys aged 6 years and two males aged 23 years were used to represent the young and aged groups, respectively. They were used as donors of bone marrow, mesenteric lymph, PBMC, and spleen samples for single‐cell sequencing. Peripheral blood samples from three young and aged rhesus monkeys were used for follow‐up validation experiments. In addition, seven aged (males aged 24–26 years) and six young (four males and two females aged 6–8 years) monkeys were used as donors of PBMCs for qPCR analysis targeting GZMB^+^ CD8^+^ T cells. All rhesus monkeys were provided by the State Key Laboratory of Primate Biomedical Research. The monkeys were individually caged and raised at a facility with humidity levels ranging from 30–70%, temperatures ranging from 18–26°C, and 12‐h cycles of light and dark. All procedures were approved by the Institutional Animal Care and Use Committee of Kunming University of Science and Technology (authorization codes: LPBR202101001, KUST202401006) and were executed in accordance with the Guide for Care and Use of Laboratory Animals (National Research Council, 2011).

### 2.2 Isolation of Immune Cells from Multiple Tissues and Organs of Rhesus Monkeys

Peripheral blood samples derived from young and aged rhesus monkeys were collected in heparinized Ficoll–Hypaque density solutions. The samples were then processed using standard density gradient centrifugation to isolate the PBMCs [19]. Bone marrow samples were derived and the cells were dissected by using standard operating procedures [20]. The mesenteric lymph white adipose tissues were dissected, washed in PBS, cut into small fragments with scissors, and then digested for 30 min at 37°C in 1 mg/mL collagenase II (Cat# V900892, Merck Millipore) and collagenase IV (Cat#17104019, Thermo Fisher Scientific). The spleen samples were dissected and splenocytes were isolated using the mechanical dissociation protocol [8]. All cell suspensions were filtered through a 40 µm strainer.

### 2.3 Single‐Cell RNA Sequencing Analysis

The single‐cell suspensions obtained from the four tissues and organs of each monkey were subjected to droplet‐based massively parallel single‐cell RNA (scRNA‐seq) sequencing using the Chromium Single Cell 3' Reagent Kit according to the manufacturer's instructions (10× Genomics). Briefly, the cell suspensions were loaded at 800 cells/µL to capture 10,000 cells. The 10× Chromium Controller generated “Gel bead in Emulsion” (GEM) droplets, where each cell was labeled with a specific barcode, and each transcript was labeled with a unique molecular identifier (UMI) during reverse transcription. The barcoded cDNA was isolated using a Dynabeads MyOne SILANE bead cleanup mixture. The cDNA was amplified by PCR and purified via SPRI bead cleanup. This consisted of fragmentation, end repair, A‐tailing, adaptor ligation, and sample index PCR, according to the manufacturer's instructions. Libraries were sequenced on a NovaSeq S4 (300 cycles) flow cell. The output BCL files generated by the NovaSeq platform were converted to FASTQ files using bcl2fastq (version 2.20) and embedded in the Cell Ranger software (version 6.0.1). The FASTQ files of each sample were converted to gene expression matrices using the Cell Ranger pipeline, which is based on references from Macaca mulatta (Rhesus monkey) (Mmul_10.102), with default settings. The output expression matrix was handed to the Seurat [21] (version 4.0.3) pipeline for filtering and analysis. We obtained a total of 240,457 cells. Cells expressing <200 or >4000 unique genes or with greater than 5% of genes of mitochondrial origid and genes presented in less than three cells were removed. After filtering, a total of 205,202 cells were retained. There were 37939, 47864, 55246, and 64153 cells obtained from the bone marrow, spleen, PBMC, and mesenteric lymph samples, respectively. In subsequently subtypes analysis of CD8^+^ T, cells with low expressing of CD8‐associated genes were further removed.

### 2.4 Integration and Clustering of scRNA‐seq Data

The raw count global scaling normalization method was implemented using the “Normalize Data” function with the “Log Normalization” method. Highly variable genes were identified using the function “Find Variable Features”. The “Find Integration Anchors” function was used to find a set of anchors between a list of Seurat objects. These anchors were subsequently used to integrate our 16 samples using the “Integrate Data” function. The “Scale Data” function was used for the z score conversion of the value of gene expression [22]. The PCA dimensionality reduction was then run against the integrated data by using the function “Run PCA”. Louvain clustering and uniform manifold approximation and projection (UMAP) [23] were then utilized for dimensional reduction. Finally, the function “Find Clusters” was used to identify clusters of cells by using the shared nearest neighbor (SNN) modularity optimization‐based clustering algorithm. The same analysis was then performed for subtypes identification.

### 2.5 Senescence‐Associated Gene Set Score

We collected several publicly reported gene sets associated with aging and performed aging‐related scoring, including: Aging‐associated Secretory Inflammatory Gene Score (ASIG) [24], CellAge Score [25], Inflammatory Response Score [26], Mesenchymal Drift Score (MD) [27], SenMayo Score [28], Senescence‐associated Inflammatory Response Score (SigRS) [29], Senescence Upregulated Score (SenUp) [30], AgingAtias Score [31], and Senescence‐Associated Secretory Phenotype Score (SASP) [26]. All scores were calculated using the “AddModuleScore” function in the “Seurat” package. The results were visualized using density distribution plots or lollipop plots, and statistical significance was assessed using the Wilcoxon rank‐sum test implemented in the “ggpubr” package.

### 2.6 GZMB Expression Analysis in GTEx and DISCO Database

GZMB expression was analyzed using both bulk and single‐cell transcriptomic data. Bulk RNA‐seq data from the GTEx database [33] were used to examine GZMB expression across human tissues, with TPM‐normalized values visualized. Single‐cell transcriptomic data for human blood and bone marrow were obtained from the DISCO database [34], and samples were stratified by donor age. Normalized GZMB expression was visualized using boxplots.

### 2.7 hUSI Analysis

The human universal senescence index (hUSI) [25] was used to calculate whether a cell is a senescent cell. This analysis is developed as a transcriptome‐based scoring method to quantify cellular senescence, in which a comprehensive dataset of 770 samples encompassing 34 cell types and 13 senescence‐inducing conditions was systematically integrated and normalized and used as training set to learn universal senescence signatures based on one‐class logistic regression (OCLR) model.

### 2.8 Correlation Between GZMB Expression and Senescence Score

Based on the Senescence‐Associated Gene Set Score, the correlation between GZMB expression and conventional aging‐related scores was further analyzed. Specifically, the cor.test function from the stats base R package was employed to perform the correlation test, and the results were visualized using dot plots and bar plots.

### 2.9 Identification of Differentially Expressed Genes (DEGs)

DEGs were identified using the Seurat function “FindAllMarkers” via the “Wilcoxon rank‐sum test”. Differential expression was performed between cell ontology groups and resulted in a list of differentially expressed genes (|log_2_FoldChange| > 0.25, *p* values < 0.05).

### 2.10 Enrichment Analysis

The “enrichKEGG” and “enrichGO” functions of the software package, clusterProfiler [35] (version 4.0.2) were used for Gene Ontology (GO) (http://www.geneontology.org) analyses and Kyoto Encyclopedia of Genes and Genomes (KEGG) (https://www.genome.jp/kegg/) pathway analyses.

### 2.11 Treatment of PBMCs with GZMB

rh‐Granzyme B (2906‐SE, RD) was activated with rm‐Cathepsin C (2336‐CY, RD) at a ratio of 10:1 (µg/mL) in an incubator at 37°C for 4 h. One million PBMCs were stimulated with activated rh‐Granzyme B (0.5 ng/µL) for 24 h in RPMI 1640 medium (11879020, Gibco), which contained IL2 (50 IU/mL, 202‐IL‐010/CF, RD), penicillin/streptomycin (15140122, Thermo Fisher Scientific), and 10% FBS (10099141C, Gibco). This was performed in a 35 mm culture dish at 37°C with 5% CO_2_. The PBMCs were then collected and analyzed via qPCR.

### 2.12 qRT‒PCR Analysis

Total RNA from the PBMCs was extracted using TRIzol (Invitrogen, US) according to the manufacturer's instructions. Approximately 1 µg of RNA was reverse transcribed into cDNA using the miRcute Plus miRNA qPCR Detection Kit (TIANGEN, China). qPCR was subsequently performed using SYBR Green PCR master mix (Roche, Switzerland). The relative expression of mRNA was normalized to that of β‐actin and calculated using the 2^−ΔΔCt^ method. The PCR primer sequences are listed in Table S1.

### 2.13 Flow Cytometry

A total of 0.5×10^6^ cells were stained with fluorescence‐labeled antibodies, followed by incubation with antibodies during 30 min at 4°C. For intracellular staining, Fixation/Permeabilization Solution Kit (BD, cat# 554714) was used. Information about antibodies used for flow cytometry is shown in Table S2. Flow cytometry was performed using the flow cytometer FACS Calibur (FACSAria II, BD, US) according to the manufacturer's instructions, and the data were analyzed using the Flowjo VX software.

### 2.14 TF‐Motif Analysis

The transcription factor (TF) regulons of the BHLHE40 CD8^+^ T cells were identified using the pySCENIC [36] (version 0.11.2) workflow with default parameters (https://github.com/aertslab/pySCENIC). TFs from hg38 were downloaded from RcisTarget [37] for use as a reference. TF networks and their corresponding target genes were visualized by Cytoscape [38] (https://cytoscape.org).

### 2.15 RNA‐Scope in Situ Hybridization

RNA‐scope in situ hybridization (*ish*) multiplex version 1 was performed according to the Advanced Cell Diagnostics (ACD) instructions. Slides of mesenteric lymphatic tissue were removed from the cryostat and immediately transferred to cold (4°C) 10% formalin for 15 min. The tissues were then dehydrated in 50% ethanol for 5 min, 70% ethanol for 5 min, and 100% ethanol for 10 min at room temperature. The slides were air‐dried briefly, and boundaries were drawn around each section using a hydrophobic pen (ImmEdge PAP pen; Vector Labs). When the hydrophobic boundaries had dried, the protease IV reagent was added to each section until it was fully covered. The protease IV incubation period was optimized according to the recommendations of the ACD. The slides were briefly washed in 1× phosphate‐buffered saline (PBS, pH 7.4) at room temperature. Each slide was then placed in a prewarmed humidity control tray (ACD) containing dampened filter paper. A mixture of Channel 1 and Channel 2 probes (using a 50:1 dilution, as directed by ACD due to the use of stock concentrations) was then pipetted onto each section until it was fully submerged. This process was performed on one slide at a time to prevent evaporation and drying of the sections. The humidity control tray was placed in a HybEZ oven (ACD) for 2 h at 40°C. All the experiments involved a target of interest in Channel 1 in combination with a BHLHE40 (Gene: *BHLHE40*, Species: Rhesus Monkey, Target Region [Base Pairs(bp)]:307‐1187, Catalog number:185051‐C1, RNAscope Probe ‐Mmu‐BHLHE40‐C1 probe); CD8 (Gene: *CD8*, Species: Rhesus Monkey, Target Region [Base Pairs(bp)]: 378–2232, Catalog number: 481881‐C2, RNAscope Probe‐ Mfa‐CD8A‐C2 probe). BHLHE40 probe in Channel 1 and a CD8 probe in Channel 2. Following probe incubation, the slides were washed twice in 1× RNA‐scope wash buffer and then returned to the oven for 30 min after being submerged in AMP‐1 reagent. The washes and amplification steps were then repeated with AMP‐2 and AMP‐3 reagents, with incubation periods of 30 and 15 min, respectively. TSA‐Plus fluorescent dye (TSA‐Plus FITC, TSA‐Plus Cy3) was then added for 30 min; the slides were then washed twice in RNA‐scope wash buffer. The slides were subsequently incubated with 1:1000 DAPI in 0.1 M PB for 1 min before being washed, air‐dried, and cover‐slipped with Prolong Gold Antifade mounting medium.

### 2.16 Knockdown of the Transcription Factors of *BHLHE40* in Human CD8^+^ T Cells

Human CD8^+^ T cells were obtained from BLUEFBI (Shanghai, China), and all the experimental protocols were approved by the Ethics Committee of Kunming University of Science and Technology (authorization code: KMUST‐MEC‐208). The CD8^+^ T cells were cultured at 37°C in 5% CO_2_ for 6 h in the medium provided by the vendor. AD (adenovirus)‐BHLHE40‐shRNA was then used, with a titer of 1×10^9^ PFU/mL, to infect 5 × 10^5^ human CD8^+^ T cells in 24‐well plates (MOI = 200) for 4 h. AD‐BHLHE40‐shRNA was constructed by Hanbio Biotechnology Co., Ltd. (Shanghai, China). Next, 1 mL of fresh medium was added, and the samples were cultured at 37°C in 5% CO_2_. The medium was replaced every 16 h. Human CD8^+^ T cells were collected after being cultured continuously for 24 h.

The total RNA of the human CD8^+^ T cells was then extracted using TRIzol (Invitrogen, USA) according to the manufacturer's instructions. Approximately 1 µg of RNA was reverse transcribed into cDNA using the miRcute Plus miRNA qPCR Detection Kit (TIANGEN, China). qPCR was then performed using SYBR Green PCR master mix (Roche, Switzerland). The relative expression of mRNA was normalized to that of β‐actin and calculated using the 2^−ΔΔCt^ method. The PCR primer sequences are listed in Table S1.

### 2.17 Pseudotime Analysis

Monocle 3 [39] (version 1.0.0) was used to infer the cell differentiation trajectories of the CD8+ T cells; for each cell, the coordinate and cell type from the original UMAP were retained. First, a principal graph was constructed using the “learn_graph” function. The cells were then ordered according to their development with the pseudotime trajectory using the “order_cell” function. Dynamic genes along the development trajectory were identified by using the “DifferentialGeneTest” function.

### 2.18 Immunofluorescence Staining

Immunofluorescence staining was conducted as previously described [40]. Briefly, 10 µm thick OCT‐embedded tissue sections were rinsed with distilled water, permeabilized with 0.4% Triton X‐100 for 30 min and washed again in PBS three times. Then, the sections were incubated with blocking buffer (10% donkey serum in PBS) at room temperature for 1 h, incubated with primary antibodies overnight at 4°C, and then with fluorescence‐labeled secondary antibodies at room temperature for 1 h. The sections were counterstained with Hoechst 33342 (Thermo Fisher, H3570, 1:1000) to visualize the nuclei. The slides were then examined using a Leica TCS SP8DIVE confocal microscope. The antibodies used for immunofluorescence staining in this study were as follows: anti‐CD8 (Zenbio, 381099, 1:100), anti‐CD44 (Abcam, ab254530, 1:200), anti‐CCL5 (Antibody, DF7427, 1:200), anti‐IL7R (Antibody, DF6362, 1:200), anti‐CD19 (Santa Cruz, sc19650, 1:100), anti‐TCL1 (Antibody, CY8823, 1:100), and anti‐PDCD4 (Abways, CY6936, 1:100). The secondary antibodies used were as follows: goat anti‐rabbit AF488 (Abcam, ab150077, 1:500), donkey anti‐rabbit AF647 (Abcam, ab150075, 1:500), and rabbit anti‐mouse‐AF555 (Abcam, ab150126, 1:500).

### 2.19 GO Enrichment Analysis

Gene Ontology (GO) enrichment analysis was performed using the R package clusterProfiler [citation], with statistical significance evaluated via the hypergeometric test. Terms with a p‐value < 0.05 were considered significantly overrepresented in the input gene set.

### 2.20 Statistical Analyses

**FIGURE 1:**
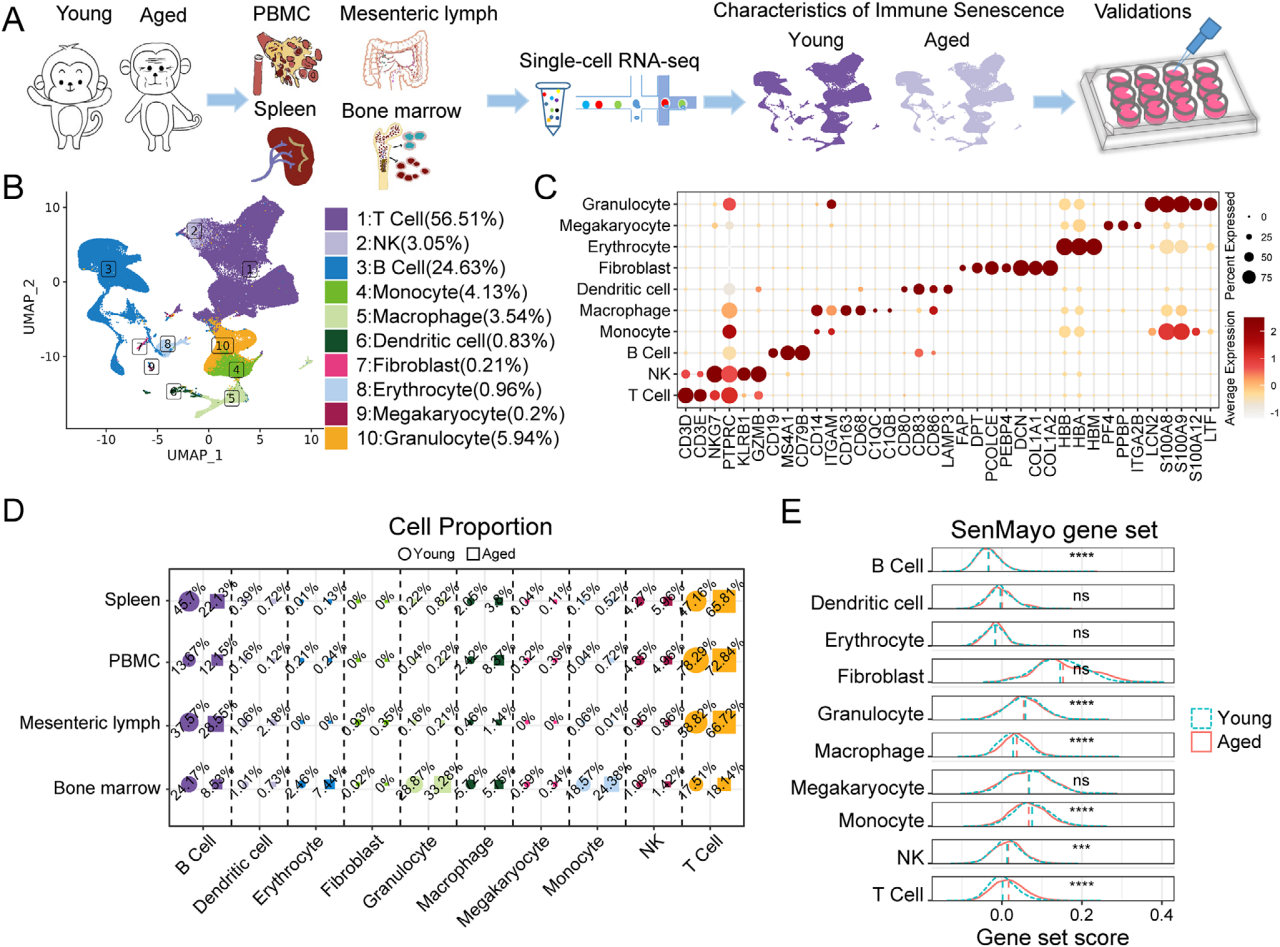
Single‐cell atlas of multiple immune tissues and organs in young and aged rhesus monkeys. (A) Flowchart of single‐cell analyses of the immune system. (B) UMAP plots showing different types of cells (205,202 cells) from four immune tissues and organs; Each dot represents a single cell with color coded cell type. (C) Dot plots showing the expression of canonical markers in various cell types. Individual bubbles show the fraction of cells expressing the corresponding gene (circle size) and the mean expression level of the gene (colour). The horizontal axis shows the list of feature genes and the vertical axis shows cell annotation. (D) Proportion of major cell types across four immune tissues. Circles represent the young and squares represent the aged groups. Marker size reflects relative abundance, and colors correspond to cell type annotations. (E) Ridge plots of representative gene set scores across immune cells in young and aged groups.

The Fisher test in the “R ggsignif” software package (version 0.6.3) was conducted to compare the changes of the cell ratios among different cell types in Figure 1C, Figure 5C, Figure 6C, and Figure S3C. Unpaired Student's t tests were used for normally distributed experimental data, such as flow cytometry and qPCR data, and normality was assessed using the Shapiro‐Wilk test before parametric tests were applied.

## 3 Result

### 3.1 Single‐Cell Transcriptomic Landscape of Four Immune Tissue and Organ Samples From Young and Aged Rhesus Monkeys

A comprehensive cell atlas depicting the cellular and systemic adaptations to aging is shown in Figure 1. In this study, we analyzed samples obtained from hematopoietic tissue (bone marrow), immune lymph tissue (mesenteric lymph), peripheral blood mononuclear cells (PBMCs), and immune organs (spleen) from young (6 years old) and aged (23 years old) male rhesus monkeys (Figure 1A). We successfully generated scRNA‐seq profiles of 205202 cells from the monkeys, and subsequently identified 10 clusters on the basis of the expression of the canonical markers (Figure 1B,C), which in the published reference [4, 7, 41, 42]. The corresponding abbreviation legend is shown in Table S3. Next, we quantified the cell proportion of each cluster within each tissue and organ for the young and aged monkeys (Figure 1D). During aging, B cells demonstrate a significant decrease across four immune organs, with the most pronounced changes observed in bone marrow, while macrophages exhibit widespread upregulation (except in bone marrow) with the most pronounced changes observed in peripheral blood mononuclear cells (PBMCs). Furthermore, in the aged group, we observed a significant of the SenMayo score, SASP score, MD score, inflammatory response gene set score, SigRS score, SenUp score, and hUSI score in most immune cells (Figure 1E, Figure S1F,G). These findings revealed that aging is accompanied by both shifts in cell composition and widespread transcriptomic alterations.

### 3.2 The Transcriptomic Consistency and Heterogeneity Across Tissues and Cell Types During Aging

Cross‐tissue aging analysis reveals a systemic elevation of SenMayo scores in aged rhesus monkeys (Figure S1A), with the bone marrow showing the highest number of DEGs, indicating pronounced genome‐wide transcriptomic remodeling (Figure S1B,C). Specifically, we identified a conserved set of 47 upregulated genes enriched for pro‐inflammatory and senescence‐associated functions, including IRF1, CCL5, and GADD45A (Figure S1D). In contrast, immunoregulatory genes such as CD1C, CXCR4, and PTGER4 were concomitantly downregulated (Figure S1E), indicative of a systemic decline in immune surveillance across tissues.

**FIGURE 2:**
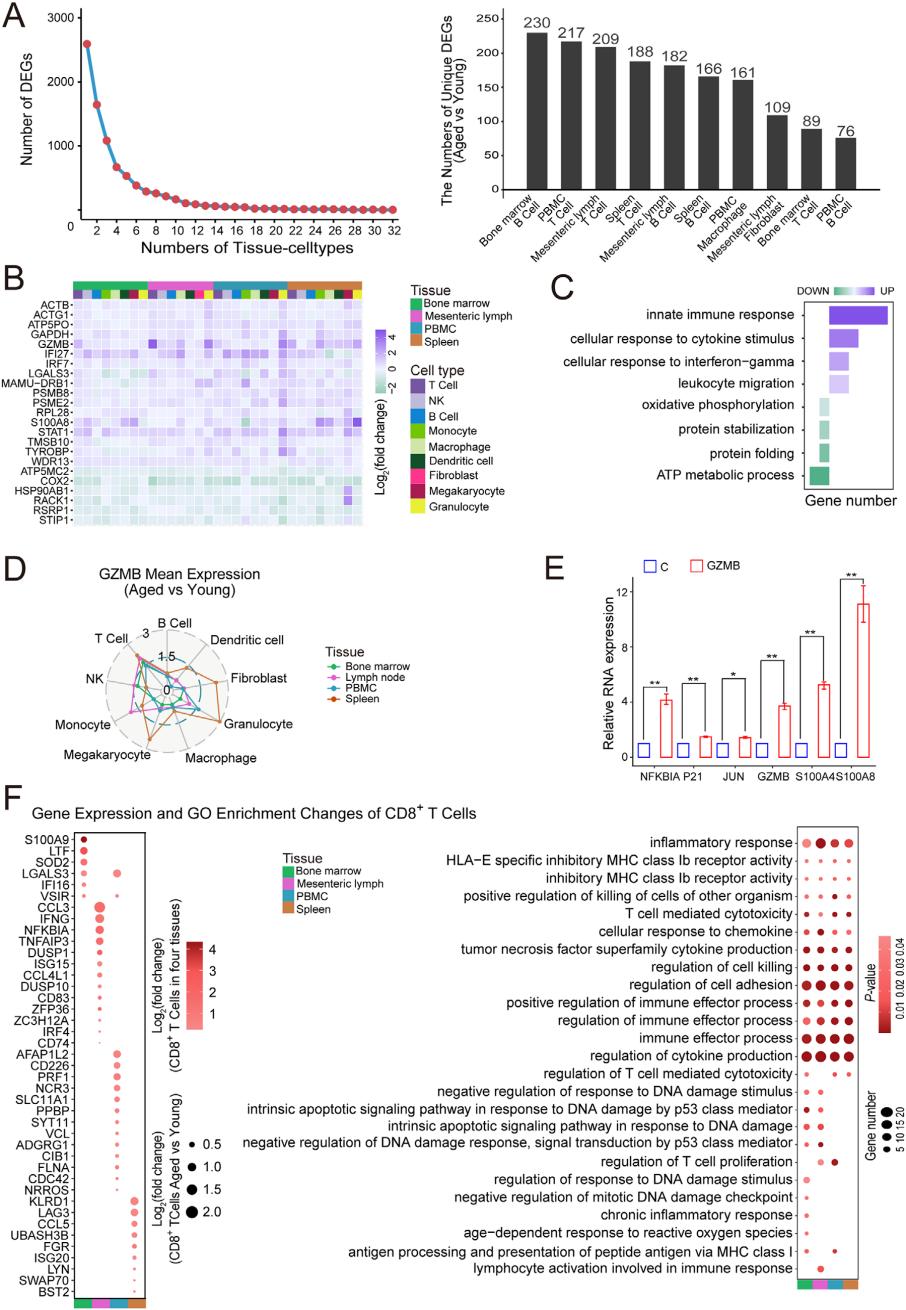
Gene expression dynamics of various tissue cell types during aging. (A) Distribution of the number of genes differentially expressed in various tissue‐cell types (left panel); The top 10 tissue cell types with the greatest changes in the number of DEGs during aging. The horizontal axis represents cell types, and the vertical axis represents the number of differentially expressed genes (right panel). (B) Heatmap showing log_2_(fold change) of genes exhibiting consensus expression changes in at least 50% of tissue‐cell types when comparing aged versus young groups. Colored blocks indicate tissue and cell type classification. (C) Pathways enrichment analysis of aging‐associated genes is shown in (B). Purple and green represent upregulated and downregulated pathways, respectively. (D) Radar Chart showing changes in *GZMB* expression across cell types between aged and young groups (*p* < 0.05). Colored lines and dots represent tissue types and cell types, respectively. (E) Exogenous GZMB treatment increased the expression of *NFKBIA*, *P21*, *JUN*, *GZMB*, *S100A4*, and *S100A8* in PBMCs. The bar chart represents the expression change in the aged group relative to the young group as evaluated by qPCR (n = 3). (F) Heatmap of DEGs (left panel) and enriched pathways (right panel) in CD8^+^‐T cell subtypes across tissues. Quantified data are presented as mean ± SEM; *p* values were determined by an independent‐samples t‐test.

We further investigated the heterogeneity and consistency of aging‐driven transcriptomic alterations across tissues and cell types. By profiling 10 major cell types from each of the four immune tissues, we analyzed 40 tissue–cell types in total and identified age‐associated DEGs in 32 of them. Most DEGs displayed strong tissue or cell‐type specificity, with only a small subset shared across all 32 types (Figure 2A), consistent with previous findings in mice [7]. Systematic comparison revealed that bone marrow B cells exhibited the greatest number of aging‐associated DEGs, followed by peripheral blood mononuclear cell (PBMC) T cells and mesenteric lymph node T cells. Despite this diversity, analysis of Figure 2A uncovered common regulatory patterns shared among multiple immune compartments. Applying stringent criteria (genes significantly altered in ≥16 of 32 tissue‐cell types), we identified a conserved core gene set including GZMB, ACTB, IRF7, and COX2, which is enriched in pathways related to innate immune response, cytokine and interferon‐γ signaling, leukocyte migration, oxidative phosphorylation, protein stabilization and folding, and ATP metabolism (Figure 2B,C). This recurrent transcriptional signature highlights both the cell‐type specificity and the conserved molecular programs that collectively define immune senescence.

We observed a pronounced accumulation of granzyme B (GZMB) across multiple immune tissues and cell types in aged monkeys (Figure 2B). GZMB expression was significantly elevated in all four immune‐related organs, showing consistent upregulation in T cells without tissue‐specific variation (Figure 2D). Treatment of young monkey PBMCs with exogenous GZMB markedly increased the expression of inflammatory and senescence‐associated genes, including NFKBIA, CDKN1A (p21), JUN, GZMB, S100A4, and S100A8 (Figure 2E), suggesting that GZMB secretion may exacerbate immune aging. Cross‐species analysis of 31 human tissue samples from the GTEx database revealed tissue‐specific GZMB expression, with the highest levels in Blood and spleen, followed by bone marrow (mesenteric lymph node data unavailable; Figure S2A). Consistently, DISCO database analysis showed age‐dependent upregulation of GZMB in human PBMCs and bone marrow cells, accompanied by a significant positive correlation with established aging scores (Figure S2B,C). Together, these findings demonstrate that increased GZMB expression closely mirrors aging status across species and immune compartments, providing new molecular evidence linking granzyme B to immune senescence.

Previous studies have reported the heterogeneity of immune tissues and organs [43]. DEG and pathway enrichment analyses in lymphoid and myeloid cells across four immune tissues revealed pronounced tissue‐specific heterogeneity during aging, particularly within CD8^+^ T cells (Figure S2D,E). Therefore, we further examined transcriptional alterations of CD8^+^ T cells among these four tissues and organs. As shown in Figure 2F, mesenteric lymph specific DEGs were enriched in inflammatory and lymphocyte activation pathways, indicating heightened inflammatory responsiveness. In contrast, bone marrow‐specific DEGs were enriched in pathways related to DNA damage repair, mitotic checkpoint regulation, chronic inflammation, and ROS response. PBMCs exhibited enhanced cytotoxic functions, such as cell killing and T‐cell proliferation, compared with other tissues, whereas the spleen showed relatively weak functional pathway enrichment. These findings highlight aging‐driven tissue specific heterogeneity in an identical cell type.

Immune cells play a pivotal role in maintaining body homeostasis [44]. We subsequently systematically investigated distinct subtypes of T cells (CD8^+^ T and CD4^+^ T cells), B cells, NK cells, and myeloid cells to investigate the fine structure of the cell subpopulations and probe their age‐dependent features sequentially.

### 3.3 Dissection of CD8^+^ T‐cell Subtypes in the Aging Environment

To analyze the aging effects at a finer resolution within CD8^+^ T cells, we reclassified CD8^+^ T cells into subtypes based on the most significantly expressed canonical marker genes. By analyzing the most significant classic marker genes in each cluster via scRNA‐seq analysis, we identified 8 cell subtypes: CD8 naïve (*CD62L* ^+^, *TCF7* ^+^, *CCR7* ^+^, *LEF1* ^+^ naïve CD8^+^ T cells), IL7R^+^ Tm (*IL7R* ^+^ memory T cells), CD8 Tcm (*CD44* ^+^ *CD62L* ^low^ *CXCR4* ^+^ central memory CD8^+^ T cells), CD8 Tem (*GZMK* ^+^ effector memory CD8^+^ T cells), cytotoxic CD8 Tex (*TOX* ^+^, *TNFRSF9* ^+^, *ENTPD1* ^+^, *EOMES* ^+^, *PD1* ^+^ cytotoxic exhausted CD8^+^ T cells), terminal CD8 Tex (*CTLA4* ^+^ terminal exhausted CD8^+^ T cells), CD8 Trm (*ANXA1* ^+^, *KLRG1* ^+^ tissue‐resident memory CD8^+^ T cells), and Temra (*CX3CR1* ^+^, *TBX21* ^+^ terminally differentiated effector memory or effector cells) (Figure S3A,B) [4, 45, 46, 47, 48, 49, 50, 51, 52, 53]. A comparative analysis of the CD8^+^ T subtypes proportions between aged and young monkeys across different tissues is shown in Figure S3C. In the aged group, a decrease in naïve T cells, IL7R^+^ Tm, Tem compared with the young group was observed across the four tissues and organs, and we observed an age‐associated increase in the abundance of terminally differentiated CD8^+^ T cells across multiple tissues in aged monkeys. Numerous studies have demonstrated a pronounced decline in naïve CD8 T cells accompanied by a contraction of IL7R transitional memory (Tm) and effector memory (Tem) subtypes, together with an accumulation of terminally differentiated or TEMRA‐like CD8 T cells across multiple tissues in both humans and nonhuman primates [54, 55, 56]. These trends are consistent with prior reports of age‐related immune remodeling.

### 3.4 Aging alters the Composition and Transcriptional Profile of CD8^+^ T‐Cell Subtypes

**FIGURE 3:**
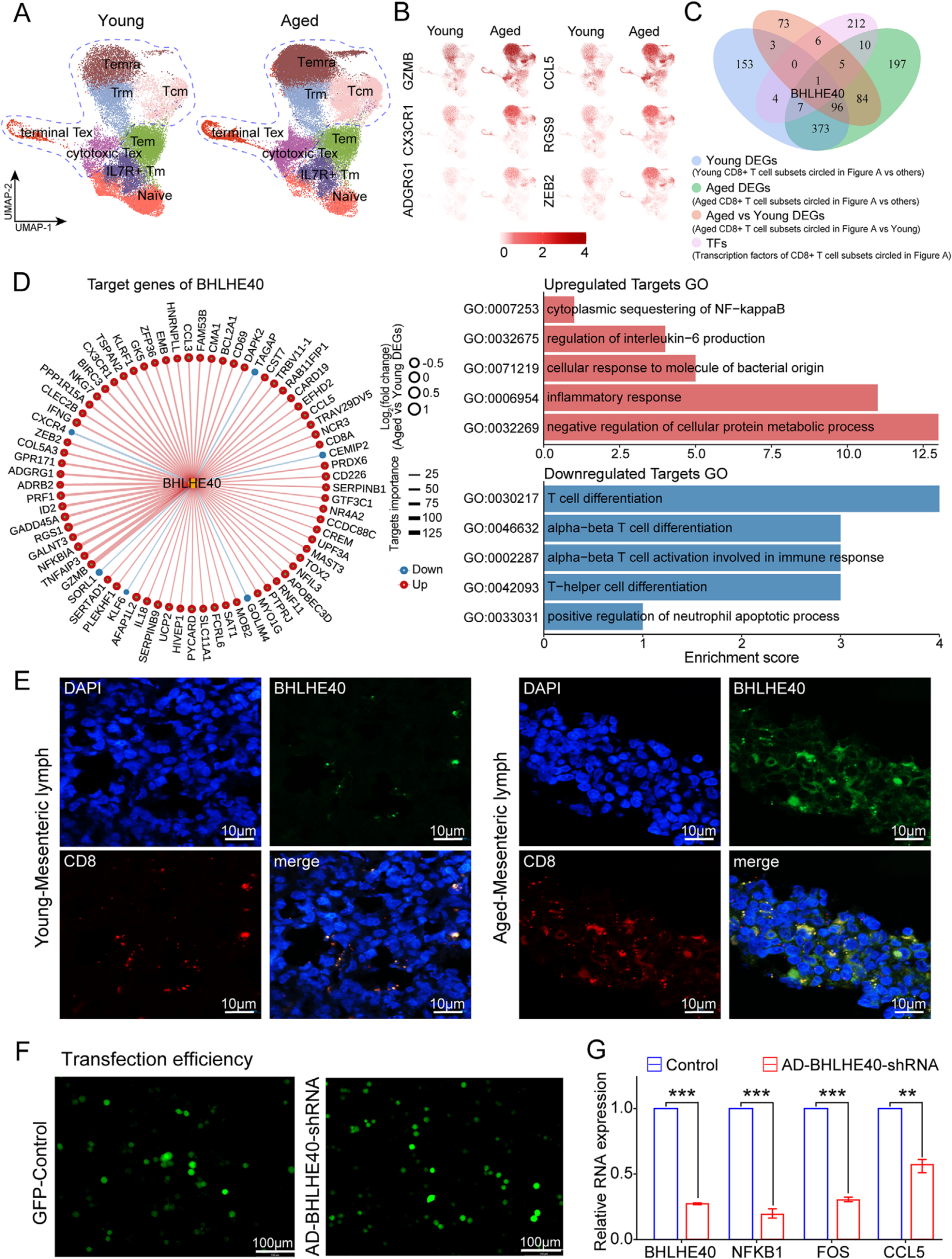
Shifts of CD8^+^ T‐Cell subtypes during aging are regulated by the transcription factor *BHLHE40*. (A) UMAP plots of CD8^+^ T‐Cell subtypes in young (left) and aged (right) groups. Each dot represents a single cell with color coded cell type, and dashed lines highlight CD8^+^ T‐cell subtypes (Tcm, Trm, cytotoxic CD8 Tex, terminal CD8 Tex, and Temra) that exhibit aging‐specific expansion. (B) Feature plots displaying expression of aging‐associated genes in young and aged CD8^+^ T‐Cells. (C) Venn diagram showing the intersection of upregulated TFs and DEGs identified by comparative analysis between or within young and aged CD8^+^ T‐Cell subtypes. (D) Network plot of *BHLHE40* and its target genes in the aging‐specific expanded CD8^+^ T‐Cell subtypes (left panel), with enriched GO terms for upregulated or downregulated pathways (right panel). (E) RNA‐scope in situ hybridization of BHLHE40 (green) and CD8 (red) in mesenteric lymphatic tissues from young and aged monkeys; Nuclei were counterstained with DAPI (blue). (F) Fluorescent images showing *BHLHE40* transfection efficiency in human CD8^+^ T cells. (G) qPCR analysis showing downregulated expression of *BHLHE40*, *NFKB1*, *FOS*, and *CCL5* following *BHLHE40* knockdown (n = 3); *p* values were determined by independent‐samples *t*‐test.

We observed an interesting phenomenon in the dissection of CD8^+^ T‐cell subtypes in the cellular aging ecosystem, where the relative abundance of 5 subtypes, including TCM, Trm, cytotoxic CD8 Tex, terminal CD8 Tex, and Temra subtypes, increased in the aged group (Figure 3A). Notably, these CD8^+^ T‐cell subtypes presented gene expression profiles commonly associated with inflammation and aging, characterized by elevated expression of genes such as *GZMB, CX3CR1, ADGRG1, CCL5, RGS9, ZEB2, S100A4, PDCD1, CCL3, GADD45A, BHLHE40*, and *IL18* (Figure 3B, Figure S3D). Furthermore, we observed significantly higher SASP, MD, Inflammatory response, SenMayo, CellAge, ASIG, AgingAtlas, SenUp, and SigRS scores in GZMB^+^ CD8^+^ T cells compared to GZMB^−^ CD8^+^ T cells, indicating that the GZMB^+^ CD8^+^ T cell subset exhibits more pronounced senescence characteristics (Figure S3E). To further validate the relevance of these transcriptional signatures, we examined *GZMB* ^+^ CD8^+^ T cells in PBMCs from aged versus young monkeys via flow cytometry. Quantification based on the Mean Fluorescence Intensity (MFI) of GZMB showed significantly higher MFI levels in the aged group compared to the young group (Figure S3F). Additionally, qPCR analysis of sorted *GZMB* ^+^ CD8^+^ T cells from PBMCs from seven aged monkeys and six young monkeys confirmed the upregulation of genes associated with inflammation and aging (*ADGRG1*, *GADD45A*, *GZMB*, *ZEB2*, and *CCL5*) in aged monkeys (Figure S3G). In addition, we conducted a systematic analysis of large‐scale transcriptomic data from human PBMC‐derived CD8^+^ T cells and observed a significant age‐dependent upregulation of GZMB expression (Figure S4A). In DISCO human datasets, CD8^+^ T cells from PBMCs showed age‐associated increases in GZMB. In bone marrow, GZMB followed a nonlinear age‐related pattern with overall higher levels in older decades, consistent with the young‐to‐old contrast observed in rhesus monkeys (Figure S4B,C). Correlation analysis further demonstrated that human CD8^+^GZMB^+^ T cells were positively associated with established aging indices, including inflammatory response score, and ASIG score (Figure S4D). We next examined GZMB expression in CD8^+^ T cells across humans, rhesus monkey, and mice. Our analysis revealed a significant age‐associated upregulation of GZMB across all species, suggesting that GZMB represents an evolutionarily conserved marker of aging. Notably, the expression levels in humans and rhesus macaques were more closely aligned, highlighting the similarity within primates (Figure S4E). To explore how the aging‐related DEGs were modulated, we analyzed the intersection of upregulated transcription factors in these CD8^+^ T‐cell subtypes (Figure 3C). Consequently, we identified the transcription factor BHLHE40, which regulated the largest number of target genes overlapping with the SASP gene set, with GZMB ranking the highest in feature importance among all predicted downstream targets. GO analysis revealed that the BHLHE40‐activated target genes were enriched in inflammation‐related and senescence‐associated pathways (e.g., NF‐κB signaling), whereas pathways involved in transcriptional regulation and T‐cell differentiation were downregulated (Figure 3D). We further observed an increased density of *BHLHE40* ^+^ CD8^+^ T cells in mesenteric lymph node CD8^+^ T cells from aged monkeys compared with young monkeys (Figure 3E). Finally, in human CD8^+^ T cells, knockdown of *BHLHE40* dramatically reduced the expression of the proinflammatory genes *NFKB1*, *FOS*, and *CCL5*, highlighting its potential conserved role in age‐related immune remodeling (Figure 3F,G). Collectively, our findings suggest that aging promotes the expansion of specific CD8^+^ T‐cell subtypes with inflammatory and senescence‐associated transcriptional features, potentially driven by *BHLHE40*‐mediated regulatory programs.

### 3.5 Cross‐Species Patterns of BHLHE40 and GZMB in Aging CD8^+^ T Cells

In human CD8^+^ T cells, BHLHE40 candidate targets, including GZMB, showed age‐associated upregulation (Figure S4F), consistent with the nonhuman primate patterns described above. Across species (human, rhesus, mouse), BHLHE40 expression itself rises with age, with nearly parallel trajectories in humans and rhesus monkeys (Figure S4G). To further evaluate whether their transcriptional variation aligns with a potential regulatory relationship, we quantified associations between BHLHE40 and GZMB across species. Significant positive correlations were observed in humans and rhesus monkeys, whereas no significant correlation was detected in mice. GZMB ranked among the strongest BHLHE40–target correlations in monkeys (Figure S4H). Together with the BHLHE40‐centered transcriptional network in CD8^+^ T cell subtypes, these cross‐species analyses support a conserved relationship between BHLHE40 and GZMB during CD8^+^ T cell aging, reflecting a broad age‐associated transcriptional shift rather than the emergence of a distinct subset.

### 3.6 Aging Induces Distinct Phenotypic Variations in CD8 Tcm Cells

**FIGURE 4:**
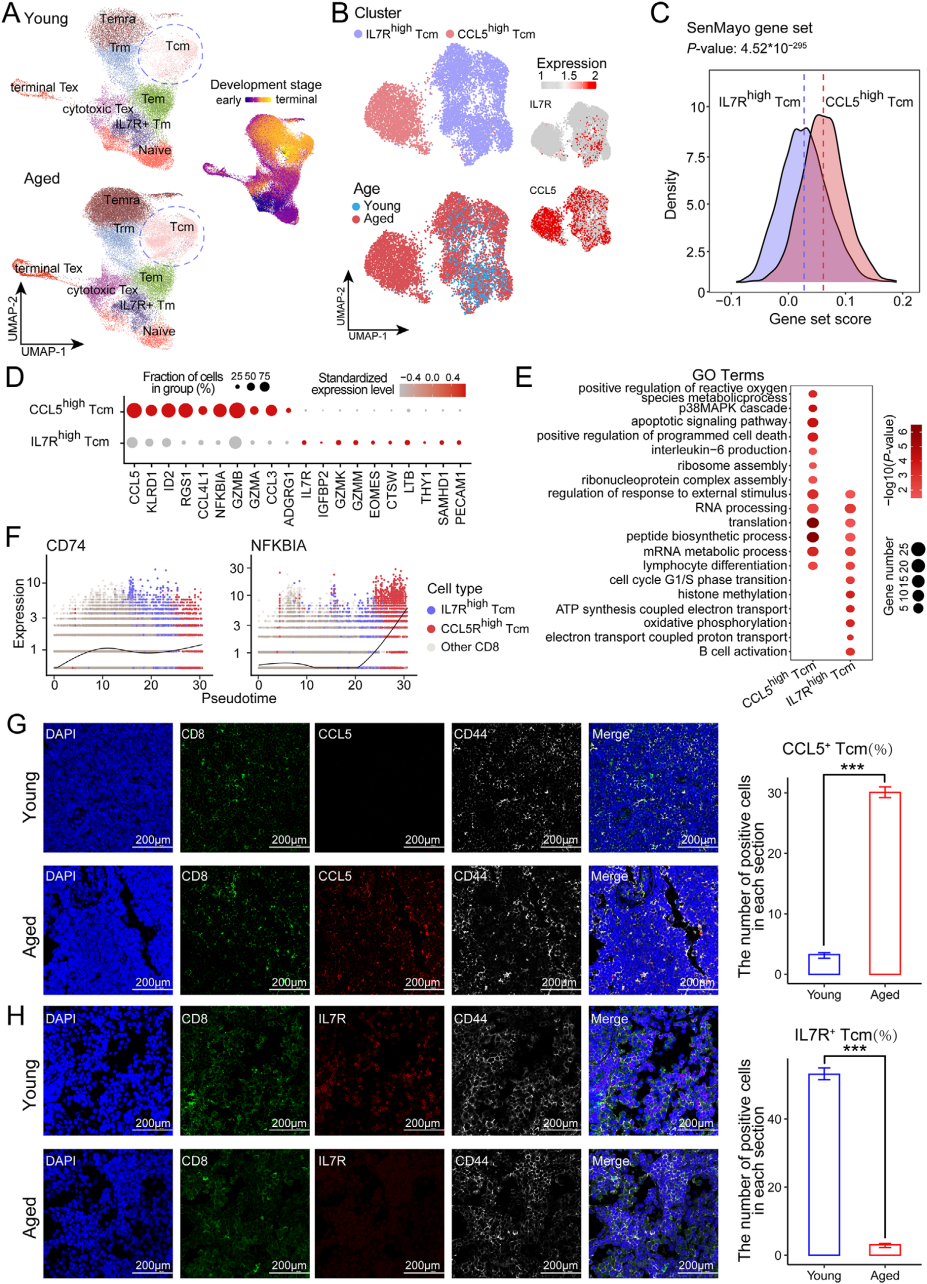
Transcriptional remodeling of CD8 Tcm cells during aging. (A) UMAP plots of CD8^+^ T cell subtypes in young and aged monkeys. Each dot represents a single cell with color coded cell type, and a dashed line highlights the increased abundance of CD8 Tcm cells with age (left panel). Pseudo‐time analysis shows the differentiation trajectory (right panel). (B) Re‐clustered CD8 Tcm cells at a finer resolution. Each dot represented a single cell with color coded cell type(top)/Age(bottom) and expression levels of IL7R/CCL5(right). Each dot represented a single cell. (C) Ridge plot showing shifts in the SenMayo gene set score between IL7R^high^ and CCL5^high^ Tcm clusters. (D) Differentially expressed genes between *CCL5* ^high^ and *IL7R* ^high^ Tcm clusters. Circle size indicates the percentage of expressing cells, and color intensity reflects the average expression level. (E) Pathway enrichment analysis of genes in (D). (F) Genes exhibiting significant pseudotime‐dependent dynamics alongside Tcm differentiation. (G‐H) Immunofluorescence analysis of *IL7R* and *CCL5* in mesenteric lymphatic CD8^+^ T cells from young and old groups. DAPI (blue), *CD8* (green), *IL7R* (red), *CCL5* (red), and *CD44* (gray); Scale bars: 200 µm. Quantified data are shown as mean ± SEM, and *p* values were determined by one‐tailed unpaired *t*‐test.

Immune memory forms the basis of vaccine‐induced protection, and memory CD8^+^ T cells constitute an important cellular component of this type of immunity. Tcm cells show stronger immune replacement and survival abilities in *vivo* than Tem cells do [57]. We found that the number of CD8 Tcm cells increased in aged monkeys (Figure 4A). To investigate this further, we reclustered the data to a fine resolution. We noted one cluster that emerged in aged cells that highly expressed *CCL5* and another cluster that was observed mainly in young cells that highly expressed *IL7R* (Figure 4B). The SenMayo score‐related genes were enriched in the *CCL5* ^high^ cluster of aged CD8^+^ T cells (Figure 4C). GO analyses were conducted to investigate the biological processes associated with the *CCL5* ^high^ and *IL7R* ^high^ cluster populations, revealing enrichment of the apoptotic signaling pathway and interleukin‐6 production in the *CCL5* ^high^ cluster (Figure 4D,E). We performed pseudotime trajectory analysis and found that the *IL7R* ^high^ cluster and the *CCL5* ^high^ cluster were primarily regulated by *CD74* and *NFKBIA*, respectively (Figure 4F). In addition, we performed immunofluorescence staining of *IL7R* and *CCL5* in CD8 Tcm cells (*CD8^+^* and *CD44^+^*) in mesenteric lymph and verified that *IL7R* was highly expressed in the young group, whereas *CCL5* was highly expressed in the aged group (Figure 4G,H). Our findings indicate that senescent CD8 Tcm cells exhibit an increased capacity for *CCL5* production and a diminished ability to generate *IL7R* during aging, which is consistent with previously described functions of *CCL5* and *IL7R* in the immune response [58, 59].

### 3.7 Dissection of Subtypes of CD4^+^ T and NK Cells in the Aging Environment

Additionally, we identified CD4^+^ T cells and divided them into four subtypes: CD4 naïve (*CCR7* ^high^ *CD69* ^low^ naïve CD4^+^ T cells), CD4 Tcm (*CCR7* ^med^ *CD69* ^high^ *CCR6* ^−^ central memory CD4^+^ T cells), CD4 Tem (*CCR6* ^+^ effector memory CD4^+^ T cells) and CD4 TRM (*ITGAE* ^+^, *RUNX3* ^+^, *CXCR6* ^+^, *PRDM1* ^+^ tissue‐resident memory CD4^+^ T cells), based on the clustering analysis of TRM cells, we identified two distinct clusters, with one cluster exhibiting significantly higher expression of cytotoxicity‐related genes (GZMA, GZMB, GZMK, GZMM, PRF1, and NKG7), leading us to further classify TRM into two subpopulations: conventional TRM and cytotoxic TRM (Figure S5A,B). In the aged group, the cell ratios for the naïve and Tcm subtypes were reduced (Figure S5C). The changes in CD4^+^ T cells we observed in the aged group were consistent with those reported in humans [45]. Next, we compared the number of differentially upregulated genes in each CD4^+^ T‐cell subtype during aging and found that the Tcm cells in the aged group presented the greatest number of upregulated genes (Figure S5D).

Aging profoundly impacts lymphocyte development and function [60]. In addition to CD8^+^ T, CD4^+^ T, and B cells, natural killer (NK) cells are also vital cellular components of the immune system. Analysis of the NK cell status revealed three separate immune states: NK1 (*NCAM1* ^+^, *IL7R* ^+^ NK), NK2 (*FCGR3* ^+^ NK), and NK3 (*IL2RB* ^+^ NK) (Figure S5E,F). The cell ratio of NK1 in the aged group was lower than that in the young group (*p* = 0.0252). In contrast, the NK2 and NK3 populations barely changed (Figure S5G). We visualized gene‐related signaling pathways for each cell type in the aged group compared with those in the young group and revealed that these pathways were activated via positive regulation of the inflammatory response and apoptotic process in NK2 cells (Figure S5H).

### 3.8 Aging Results in a Decline in Unique Naïve B Cells in the Bone Marrow

**FIGURE 5:**
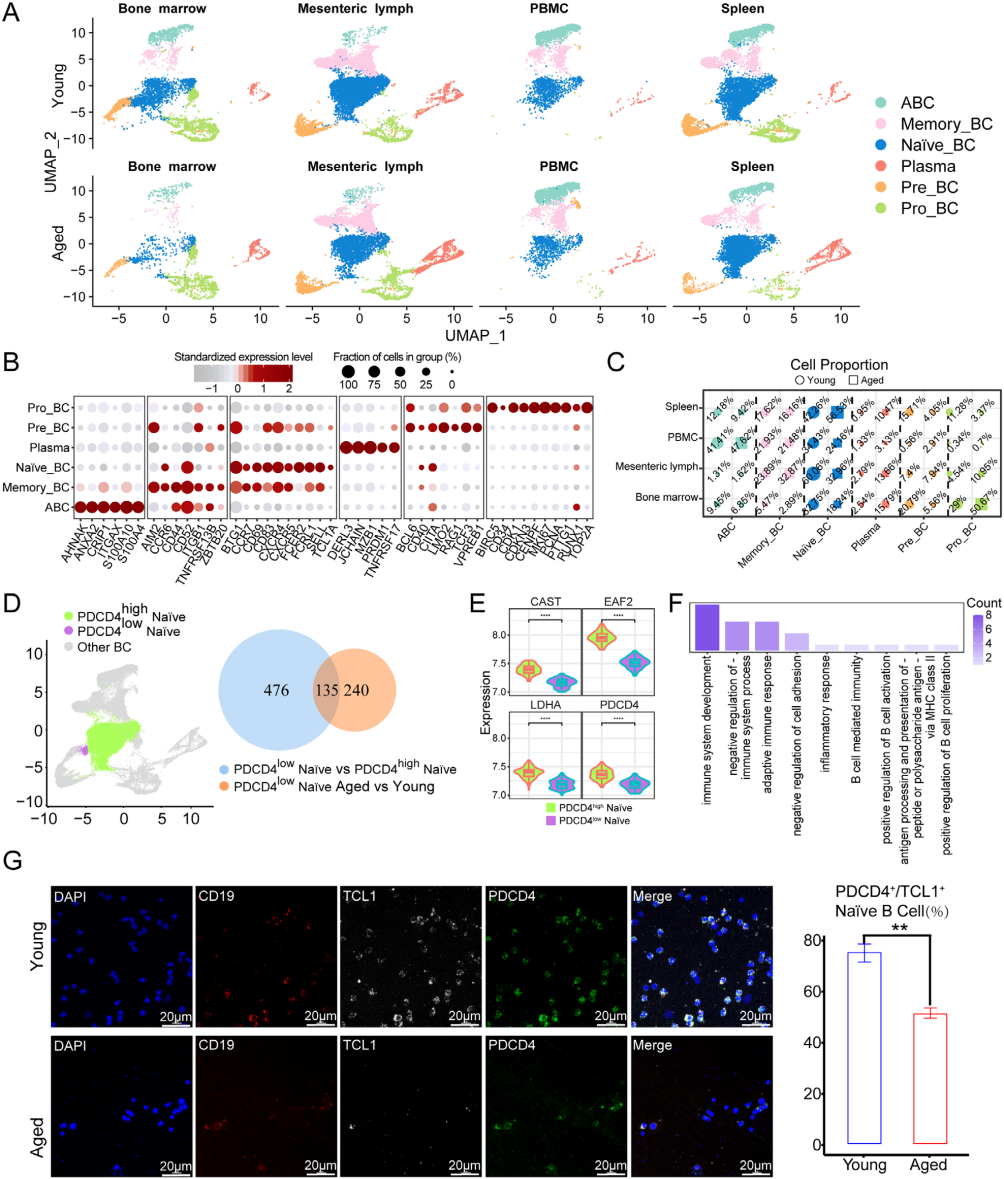
Aging manifests novel naïve B cell states in the bone marrow. (A) UMAP visualization of B‐lineage cells from four tissues and organs, colored by cell types. (B) Dot plots showing expression of canonical B‐lineage marker genes. Circle size shows the fraction of expressing cells, and color intensity reflects the mean expression level. The horizontal axis lists feature genes, and the vertical axis shows cell annotation. (C) Proportion of B‐cell subtypes across four immune tissues. Circles represent the young and squares represent the aged groups. Marker size reflects relative abundance, and colors correspond to B‐cell subtype annotations. (D) UMAP plot of bone marrow B‐lineages showing PDCD4^high^ and PDCD4^low^ naïve B cell subtype (left panel). Venn diagram showing DEGs specific to PDCD4^low^ naïve B cell subtype (476), DEGs specific to PDCD4^low^ naïve B cell subtype from old group (240), and intersecting DEGs between the two groups (135) (right panel). (E) Violin plots showing a set of top genes. Blue and red boxes represent young and aged groups, respectively, while green and purple lines indicate PDCD4^high^ and PDCD4^low^ subtypes, respectively. (F) GO enrichment analysis showing the mainly changed signaling pathways. (G) Immunofluorescence analysis of *PDCD4* in bone marrow naïve B cells from young and aged groups; DAPI (blue), *CD19* (B cell marker, red), *TCL1*(naïve B cell marker, gray), and *PDCD4* (green); Scale bars: 20µm. Quantitative data are shown as means ± SEM, and *p* values were determined by one‐tailed unpaired *t*‐test.

The number of B‐lineage cells in the bone marrow decreases during aging, which occurs at a similar rate to thymic involution but takes place somewhat late [61]. We identified 6 major B‐cell subtypes, including Pro‐B (*BIRC5* ^+^, *CD34* ^+^, *CDK1* ^+^, *CDKN3* ^+^, *CENPF* ^+^, *MKI67* ^+^, *PCNA* ^+^, *PTTG1* ^+^, *RUNX1* ^+^, *TOP2A* ^+^ B‐cell progenitors), Pre‐B (*BCL6* ^+^, *CD40* ^+^, *CIITA* ^+^, *LMO2* ^+^, *RAG1* ^+^, *TCF3* ^+^, *VPREB* ^+^ B‐cell precursors), naïve BC (*BTG1* ^+^, *CCR7* ^+^, *CD69* ^+^, *CD83* ^+^, *CXCR4* ^+^, *CXCR5* ^+^, *FCER2* ^+^, *FCRL1* ^+^, *SELL* ^+^, *TCL1A* ^+^ naïve B cells), memory BC (*AIM2* ^+^, *CCR6* ^+^, *CD44* ^+^, *CD52* ^+^, *ITGB1* ^+^, *TNFRSF13B* ^+^, *ZBTB20* ^+^ central memory B cells), ABC (*AHNAK* ^+^, *ANXA2* ^+^, *CRIP1* ^+^, *ITGAX* ^+^, *S100A10* ^+^, *S100A4* ^+^ age‐related B cells), *DERL3* ^+^, *JCHAIN* ^+^, *MZB1* ^+^, *PRDM1* ^+^, *TNFRSF17* ^+^ plasma cells (Figure 5A,B). A comparative analysis of the cell proportions in various clusters of aged to young monkeys, is presented in Figure 5C. In addition, we identified a unique naïve B‐cell subpopulation in the bone marrow that exhibited a decrease in the cell ratio and lower expression of *PDCD4* during aging, which was almost nonexistent in other tissues and organs. A total of 476 differentially expressed genes were identified between the *PDCD4* ^low^ and *PDCD4* ^high^ subtypes in the bone marrow, and there were 240 differentially expressed genes in the *PDCD4* ^low^ subtype between the young and aged groups, with 135 intersecting genes between the two groups (Figure 5D). GO analysis revealed that pathways related to B‐cell activation and proliferation were significantly altered in the PDCD4^low^ subtype within the bone marrow, with representative genes including *CAST*, *EAF2*, *LDHA*, and *PDCD4* (Figure 5E,F). Trajectory analysis positioned PDCD4^low^ naïve B cells between naïve and pre‐B stages, suggesting a transitional developmental state. The age‐related decline of PDCD4^low^ naïve B cells suggests that alterd B‐cell maturation may contribute to immune remodeling during aging (Figure S5I–K). We subsequently conducted an immunofluorescence staining experiment to validate our findings. Notably, we observed a significant decrease in the number of cells coexpressing *CD19* ^+^, *TCL1* ^+^, and *PDCD4* ^+^ in the bone marrow of aged monkeys compared with young monkeys (Figure 5G). These results demonstrated that aging is associated with reduced production of naïve B cells in the bone marrow, suggesting its potential as a senescent phenotype.

### 3.9 Heterogeneity of Myeloid Cells During Aging

**FIGURE 6:**
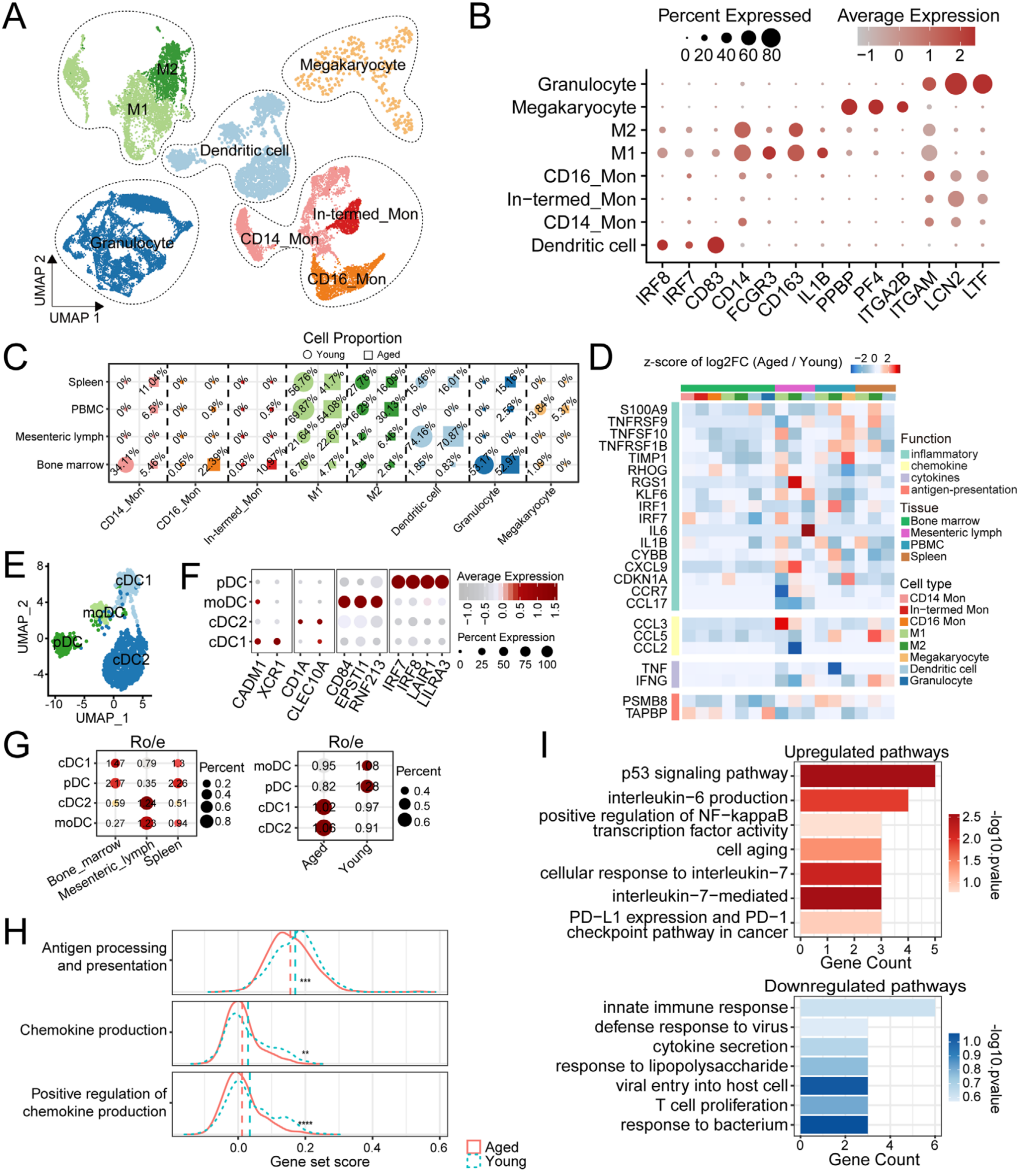
Heterogeneity of myeloid cells during aging. (A) UMAP visualization of major myeloid cell populations, including granulocytes, macrophages, monocytes, dendritic cells, and megakaryocytes. Dashed circles delineate the boundaries of each population. Each major population was subsequently replastered to resolve finer subtypes. Each dot represents a single cell, and colors denote distinct cell types. (B) Dot plots showing expression of canonical marker genes. Dot size indicates the fraction of expressing cells, and colored intensity reflects scaled expression. (C) Proportion of myeloid cell subtypes across four immune tissues. Circles represent the young and squares represent the aged groups. Marker size reflects relative abundance, and colors correspond to myeloid subtype annotations. (D) Heatmap showing fold changes (Aged vs Young) of genes involved in chemokine signaling, inflammatory responses, cytokine activity, and antigen presentation in myeloid cell populations across four tissues. Horizontal color bar indicates different tissues and cell types, and vertical color bar indicates function clusters. (E) UMAP plots showing dendritic cell subtypes. Each dot represents a single cell, colored by cell subtype. (F) Dot plots illustrating expression profiles of canonical marker genes of dendritic cell subtypes. Dot size indicates the fraction of expressing cells, and color intensity indicates scaled expression. (G) Dot plot showing Ro/e statistics and cell proportions by tissue (left) and age group (right). Numbers represent Ro/e values, and dot size corresponds to the percentage of cells within each tissue or age group. (H) Ridge plots showing gene set scores within cDC2 cells calculated with the ‘AddModuleScore’ function in Seurat. The x‐axis indicates module scores, and the y‐axis denotes gene enrichment pathways. Line colors distinguish age groups, and *p* values were determined by the Wilcoxon rank‐sum test: **p < 0.01, *** *p* < 0.001, ****p < 0.0001. (I) Bar plot showing GO enrichment results based on DEGs between aged and young cDC2 cells. All displayed pathways meet a significance threshold of *p* < 0.05.

We identified eight major myeloid cell types, including CD14‐Mon (*CD14* ^high^ *FCGR3* ^−^ classical monocytes), CD16‐Mon (*CD14* ^+/−^ *FCGR3* ^high^ nonclassical monocytes), In‐termed‐Mon (*CD14* ^+^ *FCGR3* ^+/−^ intermediate monocytes), M1 (*FCGR3* ^+^ *IL1B* ^+^ classical macrophages), M2 (*CD163* ^+^ nonclassical macrophages), *CD83^+^*, *IRF8^+^*, *IRF7^+^* dendritic cells, *ITGAM* ^+^, *LCN2^+^*, *LTF^+^* granulocytes cells, and MKs (*PPBP^+^*, *PF4^+^*, *ITGA2B^+^* Megakaryocyte) [45] (Figure 6A,B). Relative proportions of myeloid cell types in young and aged monkeys across the four tissues were displayed in Figure 6C. Myeloid cells are important for promoting antigen presentation and inflammatory activities. Therefore, we analyzed the heterogeneity in the expression of genes related to chemokines, inflammatory factors, cytokines, and antigen‐presenting factors in the four tissues and organs (Figure 6D). Our analyses observed transcriptional alterations in genes associated with chemokine signaling, cytokine production, and antigen presentation, particularly within the mesenteric lymph. To further dissect these changes, we profiled distinct dendritic cell (DC) subtypes, including plasmacytoid DCs (pDCs; *IRF7* ^+^, *IRF8* ^+^, *LAIR1* ^+^, *LILRA3* ^+^), monocyte‐derived DCs (moDCs; *CD84* ^+^, *EPSTI1* ^+^, *RNF213* ^+^), and conventional DCs (cDC1: *CADM1* ^+^, *XCR1* ^+^; cDC2: *CD1A* ^+^, *CLEC10A* ^+^) [62] (Figure 6E,F). Notably, we identified a mesenteric lymph–specific cDC2 subpopulation exhibiting and an increased proportion in aged individuals (Figure 6G). Differential gene expression (gene scoring) and GO enrichment analyses revealed that aged cDC2s exhibited enhanced inflammatory responses and reduced antigen‐presenting and chemotactic capacities (Figure 6H,I). Previous studies have reported that CD1A^+^CD172A^+^ cDC2 cells directly interact with T cells [62]. Consistent with this, our data suggest that age‐associated alteration of this subset may compromise T‐cell activation. However, the interaction between cDC2s and T cells, and the contribution of cDC2s to ageing, remain to be elucidated in the future. Collectively, these findings indicate that myeloid cells in mesenteric lymph nodes may be more susceptible to aging than those in other immune organs, with cDC2 impairment characterized by reduced antigen presentation and dampened immune activation emerging as a predominant feature.

## 4 Discussion

Owing to the challenges associated with human tissue sampling, tracking the human immunosenescence process throughout aging remains unfeasible. Previous studies have shown that nonhuman primates and humans share an abundance of immune cells and a comparative composition of cell types in corresponding organs [18], suggesting that nonhuman primates are ideal for studying human immunosenescence. Single‐cell sequencing has previously provided detailed maps of the aging process in relation to the entorhinal cortex [63], ovaries [64], arteries [65], and retina [66] of nonhuman primates. However, single‐cell maps focused on immunosenescence in humans and nonhuman primates are still lacking [18, 67]. The bone marrow serves as the primary source of various immune cells. PBMCs are distributed throughout the body via the bloodstream and play crucial roles in the immune response and regulation. Additionally, peripheral lymphoid organs encompass the spleen and mesenteric lymph nodes, serving as important sites for immune cell proliferation, maturation, differentiation, and active participation in immune responses. Therefore, it is imperative to investigate the age‐related changes occurring within these four immune tissues and organs to understand their contributions to immunosenescence. Here, we generated single‐cell transcriptomic profiles of four immune tissues and organs from young and aged rhesus monkeys, characterized the cellular landscape of immunosenescence, and identified key immunosenescence signatures, thereby filling critical gaps in understanding immune aging across the primate lifespan.

In our study, we demonstrated that GZMB is upregulated across almost all cell types in the four immune tissues and organs during the aging process. A previous study in mice identified GZMK as a potential target for addressing age‐associated dysfunctions of the immune system [40]. Both GZMK and GZMB are members of the granzyme family (GZM), and our findings in rhesus monkeys reveal distinct regulatory patterns within this family during immune aging, providing complementary insights from a primate model. The serine protease GZMB has recently been redefined as a multifunctional proinflammatory mediator rather than solely a cytotoxic effector [68]. Elevated GZMB expression has also been reported in elderly individuals with obesity, cardiovascular, and skin diseases [69, 70, 71]. Consistent with these observations, our cross‐tissue single‐cell and integrated human dataset analyses demonstrated conserved age‐associated upregulation of GZMB, underscoring its potential as a cross‐species immunosenescence marker. Although our integrated analysis incorporating human datasets showed GZMB upregulation patterns consistent with those observed in rhesus monkeys, functional validation of in human aging remains constrained by the scarcity of longitudinal samples and the limited resolution of cross‐sectional transcriptomic studies [72].

In parallel, we observed an increased abundance of CD8^+^ T‐cell subtypes with age‐associated transcriptional signatures across all immune tissues in aged monkeys, potentially regulated by the transcription factor BHLHE40. BHLHE40 has been previously implicated in T‐cell activation, cytokine production, and effector differentiation, but its role in immunosenescence has remained unclear. Our study extends its known functions by linking BHLHE40 to age‐associated transcriptional remodeling of CD8^+^ T cells in primates. These findings suggest that age‐related characteristics trigger the development of stable specific CD8^+^ T‐cell subset compositions rather than innate T‐cell signaling, and this process potentially occurs through unconventional interactions between senescent cells and the adaptive immune system [40, 73]. The concordant upregulation of BHLHE40 and its downstream targets, most notably GZMB, across species suggests that this regulatory relationship contributes to the functional polarization of senescent CD8^+^ T cells. Together, these findings provide compelling evidence that GZMB may mechanistically participate in the remodeling of immune function during aging, offering new insights into the molecular underpinnings of immunosenescence.

Elderly individuals have encountered a greater number of antigens than young individuals, resulting in an enhanced display of immunological memory. Subsequently, elderly individuals present a substantial number of specific memory effector factors within their CD8^+^ T cells, leading to memory inflation that impairs immunity and increases susceptibility to viral infections [74, 75]. It has been reported that senescent or late memory cells produce increased levels of *CCL5* in the antigen reaction. Furthermore, *IL7R* is the receptor for IL‐7, which promotes the long‐term survival and self‐renewal of Tcm by activating the JAK‐STAT5 pathway [59]. Cytokine‐activated T cells that tend to develop features of cellular senescence accumulate in aged mice [76]. Similarly, in our study, we observed an increased population of CD8 Tcm cells expressing high levels of *CCL5* and low levels of *IL7R* in aged monkeys, which may underlie the alterations in CD8 Tcm cells in adaptive immunity during aging.

In contrast to the consistent changes observed in the four immune tissues and organs, we found that the bone marrow samples from aged monkeys presented the greatest number of DEGs. Furthermore, we explored the heterogeneity of the main lymphoid and myeloid cells. Notably, CD8^+^ T cells exhibited pronounced heterogeneity in the bone marrow, and upregulated pathways related to the regulation of the response to DNA damage stimulus, negative regulation of the mitotic DNA damage checkpoint, the chronic inflammatory response, and the age‐dependent response to reactive oxygen species were exclusively observed in the bone marrow. Bone marrow is the sole source of diverse populations of B cells [61], and B cells play a crucial role in antibody secretion. In addition, we identified a unique population of naïve B cells in the bone marrow that notably decreased during aging, potentially indicating a reduction in the formation of immunoglobulin M (IgM) B‐cell receptors (BCRs). These observations collectively highlight a significant degree of tissue and cell heterogeneity during aging, with a particular emphasis on the bone marrow as potentially the most affected tissue among the four tissues and organs investigated in this study. Aging‐related heterogeneity disrupts immune homeostasis in the bone marrow by promoting oxidative stress, chronic inflammation, and biased hematopoiesis. The persistent activation of NF‐κB and dysregulation of Wnt signaling drive myeloid‐biased differentiation while impairing lymphoid lineage potential, weakening adaptive immunity. Moreover, the decline of the unique naïve B‐cell population with low *PDCD4* expression may compromise antigen responsiveness, further diminishing immune surveillance and increasing susceptibility to infections and tumor escape.

Our cross‐tissue single‐cell atlas was derived from a small, male‐only cohort (n = 4; two young and two aged), which limits generalizability. Although validations support GZMB and BHLHE40 as candidate immunosenescence biomarkers, our perturbations in vitro (exogenous GZMB; BHLHE40 knockdown) are supportive rather than demonstrably causal. In particular, the proposed relationship between BHLHE40 and GZMB in CD8^+^ T cells is based on network and cross‐species association analyses and has not been directly established. Future work will test whether BHLHE40 regulates GZMB expression/secretion and drives aging phenotypes, using longitudinal ultrasensitive plasma assays together with gain‐ and loss‐of‐function and rescue experiments in larger, sex‐balanced cohorts to strengthen mechanistic inference.

In conclusion, our data provide a comprehensive transcriptomic aging profile for major immune organs that reflects the young‐to‐aging lifespan of rhesus monkeys, providing an essential resource for researchers from diverse disciplines. Our observations of common aging patterns contribute to the elucidation of the remarkable health benefits associated with interventions that target the aging process. Importantly, these transcriptomic aging profiles offer valuable insights into potential therapeutic strategies aimed at extending longevity. Overall, this organism‐wide characterization of aging‐related dynamics holds great promise for expediting therapeutic development.

## Author Contributions

S.W., H.Y., Y.Y., and L.R. conducted biological experiments and data collection. S.W., Z.Z., B.B., Y.W., L.W., and N.Y performed transcriptomic analysis. S.W., B.B., W.J., and W.S. conceived and designed the experiments and analyses. S.W., B.B., and W.S. prepared the paper. W.J. and W.S. supervised the work.

## Conflicts of Interest

The authors declare no conflicts of interest

## Supporting information




**Supporting File 1**: advs73863‐sup‐0001‐SuppMat.docx.

**Supporting File 2**: advs73863‐sup‐0002‐FigureS1.tif.

**Supporting File 3**: advs73863‐sup‐0003‐FigureS2.tif.

**Supporting File 4**: advs73863‐sup‐0004‐FigureS3.tif.

**Supporting File 5**: advs73863‐sup‐0005‐FigureS4.tif.

**Supporting File 6**: advs73863‐sup‐0006‐FigureS5.tif.

## Data Availability

The ScRNA‐seq, bulk RNA‐seq, and CosMx SMI raw data generated in this study have been deposited in the GEO Omnibus database under accession code GSE230908, respectively. Processed scRNA‐seq and CosMx data can be explored here. Source data are provided with this paper.
