# Supplementary material for: Single‐Cell Profiling Across Immune Tissues and Organs Reveals Immunosenescence Signatures in Male Rhesus Monkeys
Source: Adv Sci (Weinh). 2026 Jan 20;13(17):e14353. doi: 10.1002/advs.202514353 (PMC13042567; doi:10.1002/advs.202514353)
Supplement: Supplementary file 1 — Supporting File 1: advs73863‐sup‐0001‐SuppMat.docx. [file ADVS-13-e14353-s003.docx]

Table S1: PCR primer sequences

| Name of primer | [Primer sequence](javascript:;) |
| --- | --- |
| S100A8-F | 5’-ATCTGGAGAAAGCCTTGAAC-3’ |
| S100A8-R | 5’-TTTGAACCAGGCATCTGC-3’ |
| S100A4-F | 5’-GCAGGACAGGAAGACACAGT-3’ |
| S100A4-R | 5’-CTTAGACAGCAACAGGGACA-3’ |
| GZMB-F | 5’-ACTGTTGAGTTGTGCGTGG-3’ |
| GZMB-R | 5’-TGGAGGCTTGCCATTTCT-3’ |
| P21-F | 5’-CTGCCGAAGTCAGTTCCTTGTG-3’ |
| P21-R | 5’-CTCTCTCACCTCCTCTGAGTGC-3’ |
| NFKBIA-F | 5’-CTGAGTCAGGACTCCCACG-3’ |
| NFKBIA-R | 5’-ACCAACCAGCCAGAAATTG-3’ |
| JUN-F | 5’-ACGTGAAGTGACGGACTGTT-3’ |
| JUN-R | 5’-TGTAGCCATAAGCTCCGCTC-3’ |
| BHLHE40-F | 5’-TCCAAGTGACCCAAAGTTG-3’ |
| BHLHE40-R | 5’-GGACAGCAAGGAGACCTACA-3’ |
| CCL5-F | 5’-GGGTAGGATAGTGAGGGGA-3’ |
| CCL5-R | 5’-CAGAGAAGAAATGGGTTCG-3’ |
| FOS-F | 5’-AGACTACGAGGCGTCATCCT-3’ |
| FOS-R | 5’-ATGCTGGAGAAGGAATCGGC-3’ |
| GADD45A-F | 5’-TTGGAGGAATTCTCGGCTGG-3’ |
| GADD45A-R | 5’-GGTCGACGTTGAGCAGTTTG-3’ |
| ZEB2-F | 5’-GAAGCTGTAGAGAGGGACAGAAG-3’ |
| ZEB2-R | 5’-ACACAGGAATTAGTCTCTGAACCAC-3’ |
| ADGRG1-F | 5’-GTCCGGCTGTGGTTTGAGAG-3’ |
| ADGRG1-R | 5’-TGGGCAGTCATTTTCCTCCC-3’ |

Table S2: Antibodies used for flow cytometry and Immunofluorescence

| Antibodies | Vendor | Catalog number |
| --- | --- | --- |
| CD8, PE | BD | 557086 |
| GZMB, AlexaFluor 647 | BD | 560212 |
| NKG7, FITC | Cell Signaling Technology | 84835S |
| anti-CD8 | zenbio | 381099 |
| anti-CD44 | abcam | ab254530 |
| anti-CCL5 | Abways | DF7427 |
| anti-IL7R | Abways | DF6362 |
| anti-CD19 | SANTA CRUZ | sc19650 |
| anti-TCL1 | Abways | CY8823 |
| anti-PDCD4 | Abways | CY6936 |
| goat anti-rabbit AF488 | abcam | ab150077 |
| [donkey](https://www.abcam.cn/products/secondary-antibodies/donkey-rabbit-igg-hl-alexa-fluor-488-ab150073.html) anti-rabbit AF647 | abcam | ab150075 |
| abbit anti mouse-AF555 | abcam | ab150126 |

Table S3: Cell types, corresponding abbreviation, and canonical lineage markers

| Cell type | Abbreviation | Markers^4,7,43,44^ |
| --- | --- | --- |
| T-lymphocyte cell | T cell | CD3E, CD3D, CD3G, CD8A, CD4, IL2RA |
| Natural killer cell | NK | NCAM1, FCGR3, GZMA, KLRB1 |
| B cell | B cell | CD19, MS4A1, CD79B |
| Monocyte cell | Mon | CD14 |
| macrophage | Mac | C1QB, C1QC, VSIG4 |
| Dendritic cell | DC | CD80, CD83, CD86, CIITA |
| fibroblast |  | MMP2, SFRP2, COL1A2, COL1A1, DCN, CLU |
| erythrocyte | Ery | HBB, HBM, HBA |
| megakaryocyte | Meg | PPBP, PF4, ITGA2B |
| granulocyte | Gra | ANPEP, ITGAM, FCGR2B |
