# Supplementary figures and images for: Single‐Cell Profiling Across Immune Tissues and Organs Reveals Immunosenescence Signatures in Male Rhesus Monkeys
Source: Adv Sci (Weinh). 2026 Jan 20;13(17):e14353. doi: 10.1002/advs.202514353 (PMC13042567; doi:10.1002/advs.202514353)

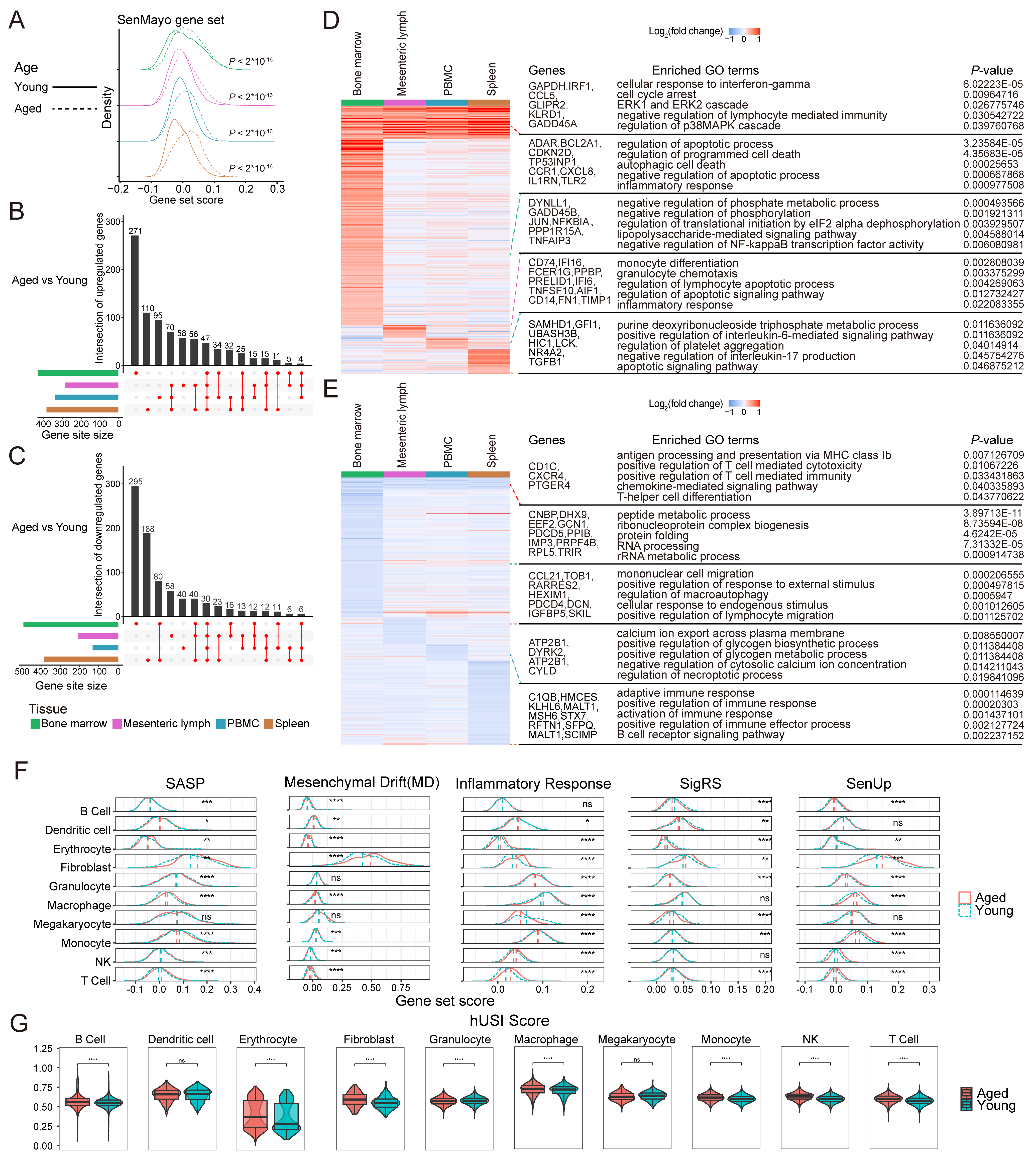

Supplement: Supplementary file 2 — Supporting File 2: advs73863‐sup‐0002‐FigureS1.tif. [file ADVS-13-e14353-s002.tif]

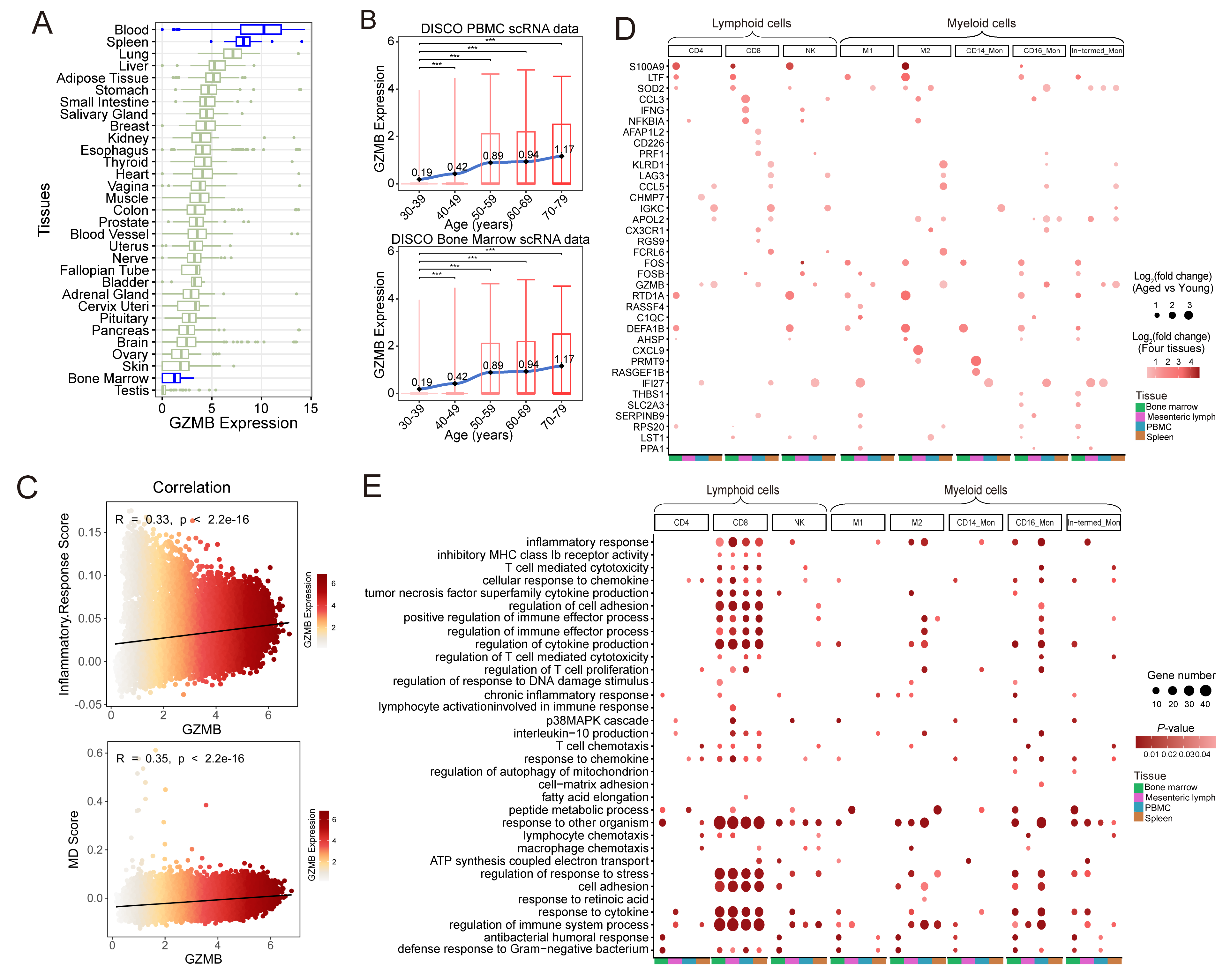

Supplement: Supplementary file 3 — Supporting File 3: advs73863‐sup‐0003‐FigureS2.tif. [file ADVS-13-e14353-s005.tif]

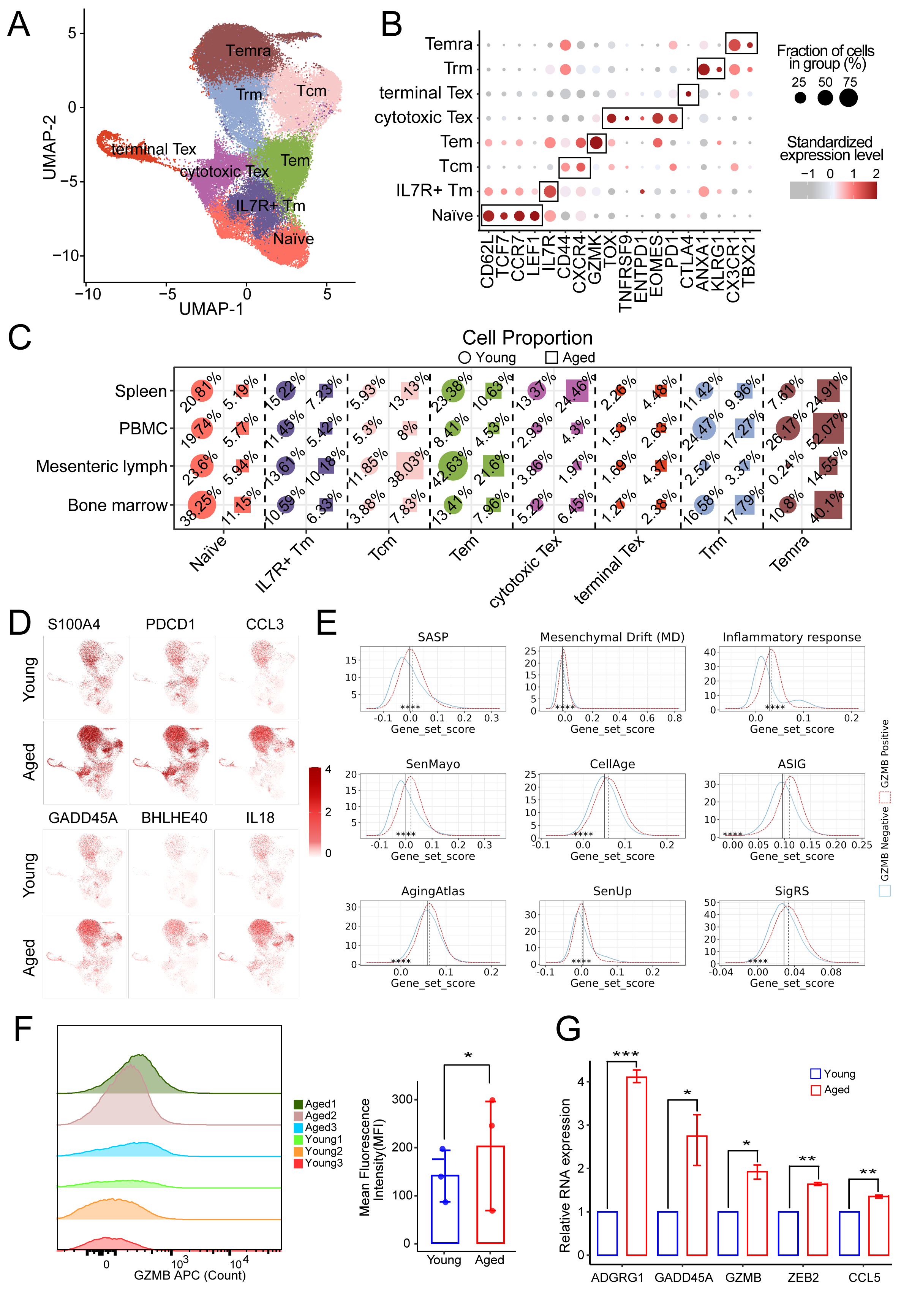

Supplement: Supplementary file 4 — Supporting File 4: advs73863‐sup‐0004‐FigureS3.tif. [file ADVS-13-e14353-s001.tif]

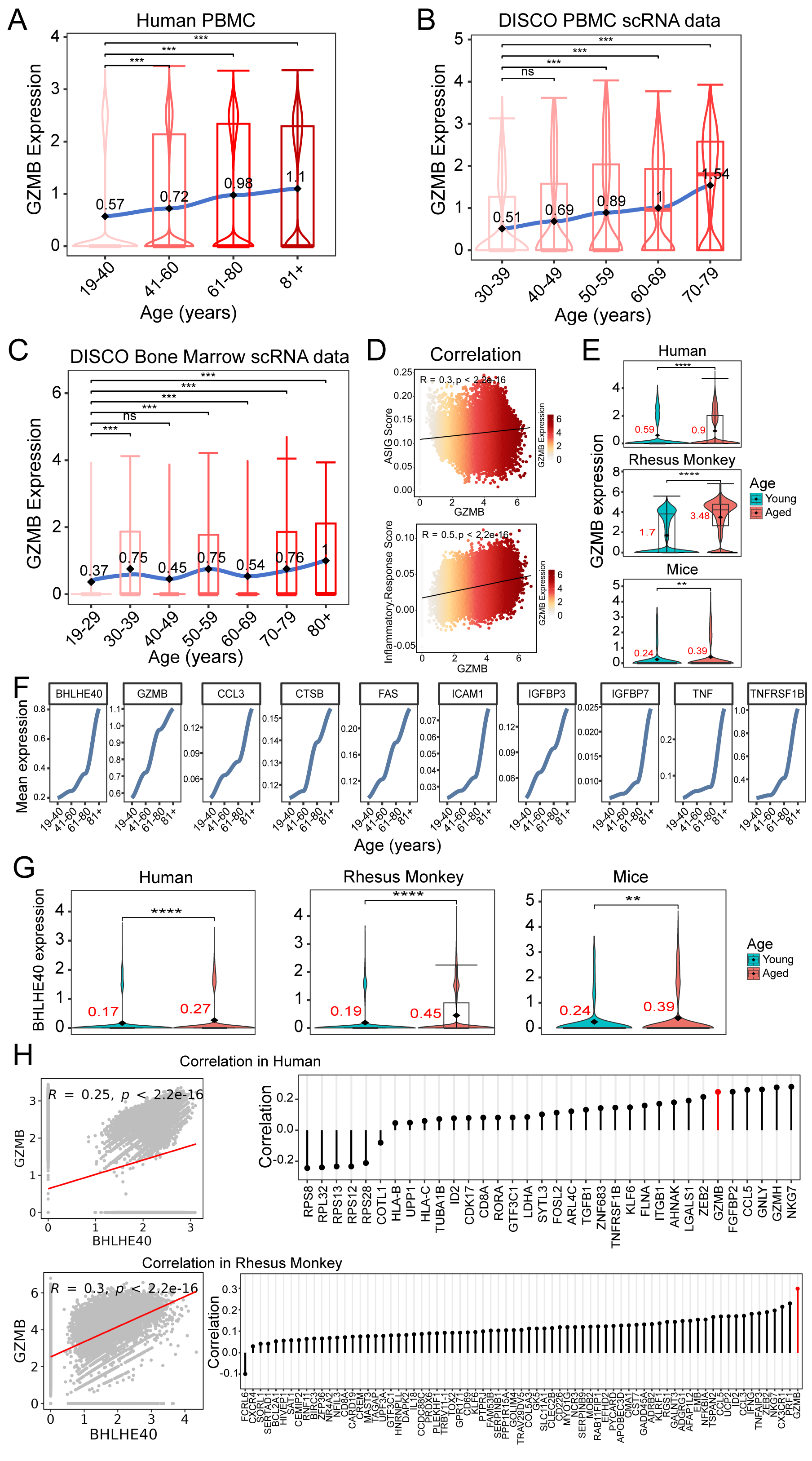

Supplement: Supplementary file 5 — Supporting File 5: advs73863‐sup‐0005‐FigureS4.tif. [file ADVS-13-e14353-s004.tif]

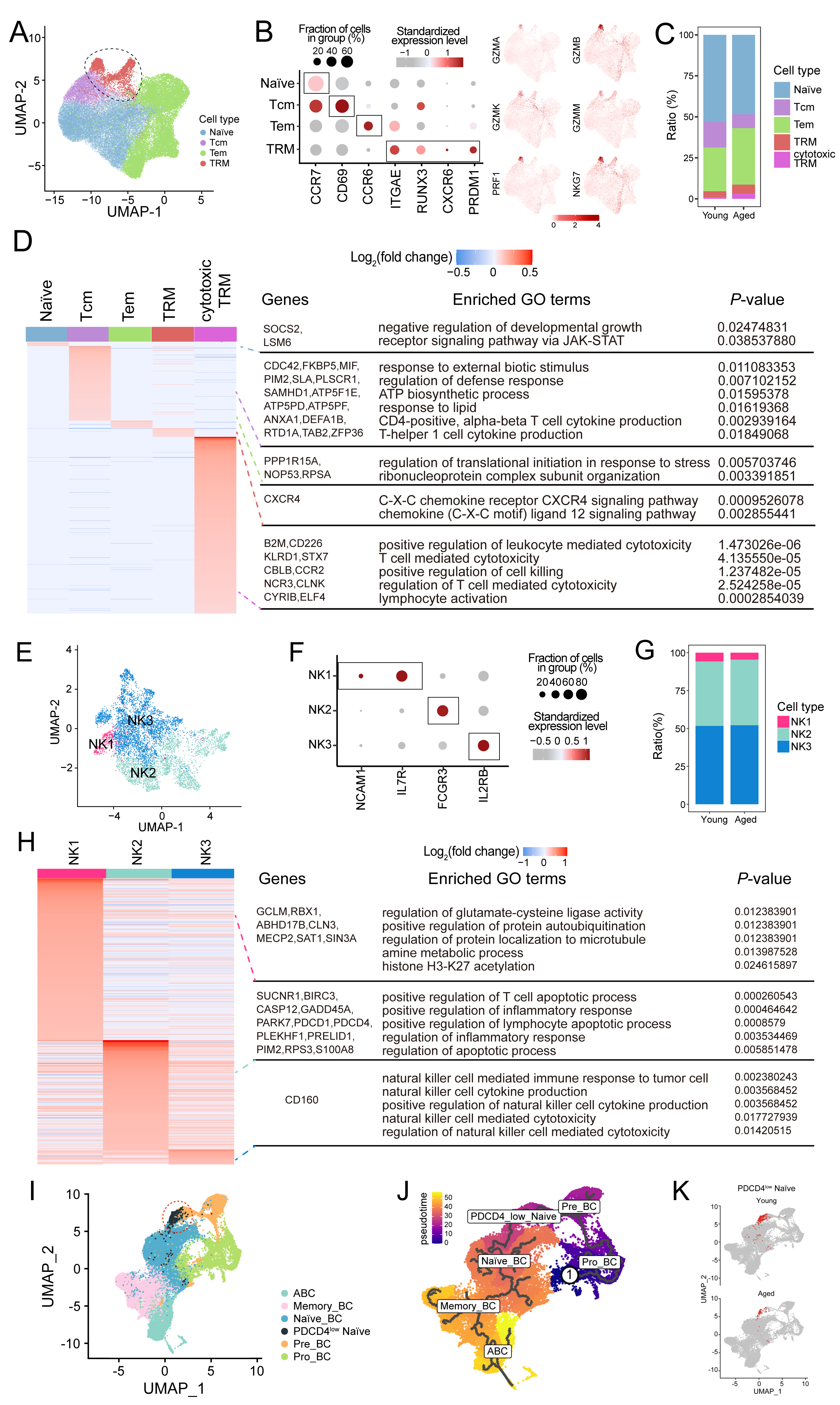

Supplement: Supplementary file 6 — Supporting File 6: advs73863‐sup‐0006‐FigureS5.tif. [file ADVS-13-e14353-s006.tif]
